# New 1,3,4-Oxadiazole Derivatives of Pyridothiazine-1,1-Dioxide with Anti-Inflammatory Activity

**DOI:** 10.3390/ijms21239122

**Published:** 2020-11-30

**Authors:** Teresa Glomb, Benita Wiatrak, Katarzyna Gębczak, Tomasz Gębarowski, Dorota Bodetko, Żaneta Czyżnikowska, Piotr Świątek

**Affiliations:** 1 Department of Chemistry of Drugs, Faculty of Pharmacy, Wroclaw Medical University, Borowska 211, 50-556 Wroclaw, Poland; teresa.glomb@umed.wroc.pl; 2 Department of Basic Medical Sciences, Faculty of Pharmacy, Wroclaw Medical University, Borowska 211, 50-556 Wroclaw, Poland; benita.wiatrak@umed.wroc.pl (B.W.); katarzyna.gebczak@umed.wroc.pl (K.G.); tomasz.gebarowski@umed.wroc.pl (T.G.); dorota.bodetko@umed.wroc.pl (D.B.); 3 Department of Pharmacology, Faculty of Medicine, Wroclaw Medical University, Mikulicza-Radeckiego 2, 50-345 Wroclaw, Poland; 4 Department of Inorganic Chemistry, Faculty of Pharmacy, Wroclaw Medical University, Borowska 211a, 50-556 Wroclaw, Poland; zaneta.czyznikowska@umed.wroc.pl

**Keywords:** pyridothiazine-1,1-dioxide, 1,3,4-oxadiazole, cyclooxygenase, molecular docking

## Abstract

Numerous studies have confirmed the coexistence of oxidative stress and inflammatory processes. Long-term inflammation and oxidative stress may significantly affect the initiation of the neoplastic transformation process. Here, we describe the synthesis of a new series of Mannich base-type hybrid compounds containing an arylpiperazine residue, 1,3,4-oxadiazole ring, and pyridothiazine-1,1-dioxide core. The synthesis was carried out with the hope that the hybridization of different pharmacophoric molecules would result in a synergistic effect on their anti-inflammatory activity, especially the ability to inhibit cyclooxygenase. The obtained compounds were investigated in terms of their potencies to inhibit cyclooxygenase COX-1 and COX-2 enzymes with the use of the colorimetric inhibitor screening assay. Their antioxidant and cytotoxic effect on normal human dermal fibroblasts (NHDF) was also studied. Strong COX-2 inhibitory activity was observed after the use of **TG6** and, especially, **TG4**. The **TG11** compound, as well as reference meloxicam, turned out to be a preferential COX-2 inhibitor. **TG12** was, in turn, a non-selective COX inhibitor. A molecular docking study was performed to understand the binding interaction of compounds at the active site of cyclooxygenases.

## 1. Introduction

Cyclooxygenase (COX) is an enzyme that catalyzes the conversion of arachidonic acid to prostanoids, which include prostaglandins, prostacyclin, and thromboxane [1]. There are two main isoforms of the COX enzyme: COX-1 and COX-2. These isoforms differ in their expression and distribution, but are similar in terms of their size, substrate specificity, and kinetics [2]. COX-1, which is expressed constitutively in most cells, is the dominant source of prostanoids that play a homeostatic role in physiological functions (such as gastrointestinal protection, platelet aggregation, and renal hemodynamics), while COX-2, which is induced by inflammatory impulses, is a more important source of prostanoid formation in inflammation and proliferative diseases, such as cancer [3,4]. Originally, COX-2 was believed to occur only in pathophysiological responses, but it is now clear that it also plays an essential role in the brain, kidney, gut, and cardiovascular systems [5]. The two cyclooxygenase isoforms are targets of nonsteroidal anti-inflammatory drugs (NSAIDs), which are competitive inhibitors of both enzymes [6]. Research has shown that both the therapeutic and side effects of NSAIDs are dependent on the same mechanism of action. It has been accepted that COX-2 inhibition is responsible for the anti-inflammatory and analgesic activity, while the ulcerogenic and renal side effects are associated with COX-1 inhibition [3,5,7]. Nowadays, we know that even selective COX-2 inhibitors related to a lower gastrointestinal risk can trigger serious cardiovascular toxicity [8]. Therefore, scientists worldwide are searching for new structures that would have a safer profile of action, while maintaining the mechanism of action as cyclooxygenase inhibitors.

Moreover, other studies show that COX inhibitors not only have anti-inflammatory activity, but also limit the production of free radicals [9,10]. Free radicals, which include reactive oxygen species (ROS) and reactive nitrogen species (RNS), are atoms or molecules with one or more unpaired electrons, which results in their high chemical activity. They can be produced in the body as by-products of the cell’s oxygen metabolism under physiological conditions and during an ongoing inflammation process. Dysfunction of antioxidant protective mechanisms leads to an imbalance in redox homeostasis in cells, the excessive production of free radicals, and, consequently, oxidative stress. Numerous studies have confirmed the coexistence of oxidative stress and inflammatory processes. Free oxygen and nitrogen radicals released by inflammatory cells lead to tissue damage. At the same time, an excess of reactive oxygen and nitrogen species and oxidative stress products may enhance inflammatory processes by modifying the expression of pro-inflammatory modulators (cytokines, chemokines, COX-2, and nuclear factor kappa B—NF-κB). It is also believed that long-term inflammation and oxidative stress may significantly affect the initiation of neoplastic transformation processes [11,12,13]. Searching for new anti-COX derivatives is also an important issue in the development of chemopreventive drugs.

The latest, and one of the most efficient, approaches in medicinal chemistry is molecular hybridization for drug development, which is based on combining pharmacophoric moieties of different biologically active substances, in order to generate a hybrid molecule with synergistic, improved affinity and efficacy, compared to the standard drug [14,15].

1,3,4-oxadiazole derivatives are five-membered ring heterocyclic compounds with a very wide range of biological activities, which makes them important construction motifs for the development of new drugs. The 1,3,4-oxadiazole ring has attracted interest in medicinal chemistry as a bioisostere for carbonyl-containing compounds, such as carboxylic acids, esters, and amides. The oxadiazole ring is used as a substantial part of the pharmacophore, which has the ability to bind with the ligand. In some cases, it acts as a flat aromatic linker that provides the appropriate orientation of the molecule [16].

These characteristics of the 1,3,4-oxadiazole ring have resulted in diverse pharmaceutical applications of these molecules in the field of medicinal chemistry. According to the literature, compounds containing the 1,3,4-oxadiazole core display a broad spectrum of biological activity, including an antibacterial [17,18], antifungal [19,20], antitubercular [21,22], antiviral [23], anticancer [24,25], and anti-diabetic [26] effect. Their analgesic [27] and anti-inflammatory effect [28,29], which often includes their mechanism of action as cyclooxygenase inhibitors [30,31,32], is particularly interesting. Moreover, a literature survey revealed that replacing the free carboxylic group in conventional NSAIDs with a 1,3,4-oxadiazole ring resulted in retained or even increased anti-inflammatory activity with reduced ulcerogenic potential [33,34,35,36]. This chemical modification has been applied to improve the safety profile of NSAIDs.

**Figure 1:**
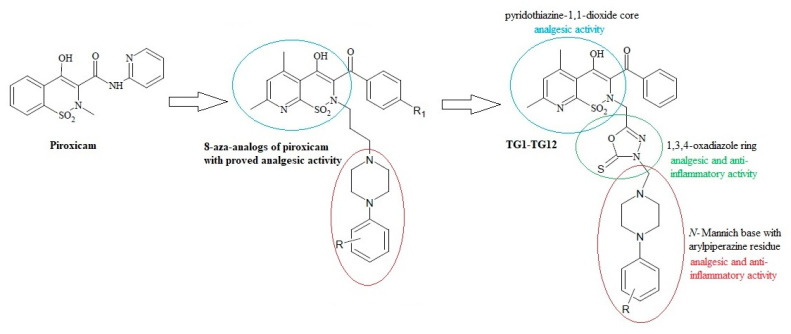
Scheme of the hybridization concept with the activity of individual structures.

On the other hand, the analgesic activity of the derivatives of pyridothiazine-1,1-dioxide designed as 8-aza-analogs of piroxicam was proven in a writhing syndrome test in mice [37] (Figure 1). In their structure, they have an arylpiperazinylpropyl moiety at position 2. Biologically active compounds containing the pharmacophoric arylpiperazine scaffold are frequently studied and have been found to possess potent anti-inflammatory activity in vitro and in vivo [38,39,40,41].

Based on this information, we decided to modify pyridothiazine-1,1-dioxide at position 2 by replacing the straight aliphatic propyl chain with the 1,3,4-oxadiazole ring. This modification can affect the stiffening of the structure of the molecule, enhance activity, and reduce toxicity.

Moreover, numerous literature reports have confirmed the multidirectional effect of the 1,3,4-oxadiazole derivatives with the structure of *N*-Mannich bases [42,43,44,45,46]. Their anti-inflammatory activity is particularly worth noting [36,47,48,49]. For this reason, our final products were designed as *N*-Mannich bases.

Motivated by the findings mentioned above, we synthesized a new series of Mannich base-type hybrid compounds containing an arylpiperazine residue, 1,3,4-oxadiazole ring, and pyridothiazine-1,1-dioxide core. The scheme of the concept is presented in Figure 1. The synthesis was carried out with the hope that the hybridization of different pharmacophoric molecules would result in a synergistic effect on their anti-inflammatory activity, especially the ability to inhibit cyclooxygenase.

The compounds were investigated in terms of their potencies to inhibit COX-1 and COX-2 enzymes with the use of the colorimetric inhibitor screening assay, and the mode of binding was characterized by a molecular docking study.

## 2. Results and Discussion

### 2.1. Chemistry

#### Synthesis of New 1,3,4-Oxadiazole Derivatives of Pyridothiazine-1,1-Dioxide

**Figure 2:**
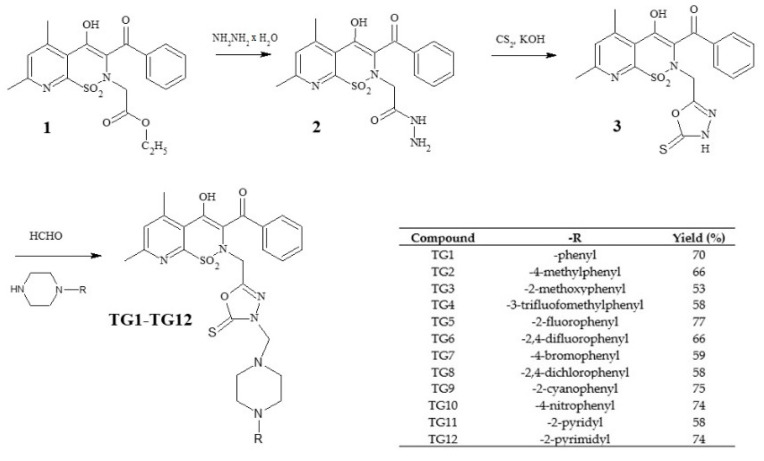
Scheme of the synthesis of new 1,3,4-oxadiazole derivatives of pyridothiazine-1,1-dioxide.

The new derivatives were obtained as a result of multistage synthesis, as shown in Figure 2. The key intermediate ethyl ester of 3-benzoyl-4-hydroxy-5,7-dimethyl-2*H*-pyrido[3,2-*e*]-1,2-thiazine-1,1-dioxide-2-acetic acid (**1**) was obtained by a few reactions, which have been described in the literature [50,51]. Ester (**1**) was easily converted into pyridothiazine-1,1-dioxide-2-acetic acid hydrazide (**2**) in a reaction with hydrazine hydrate. Hydrazide (**2**) underwent intramolecular cyclization in the presence of CS_2_, with the formation of the 1,3,4-oxadiazole derivative (**3**), which was the key substrate employed for the synthesis of the **TG1-TG12** compounds. Finally, compounds **TG1-TG12** were all prepared through a one-step Mannich reaction from structure **3**, formaldehyde, and corresponding commercially available 4-substituted-piperazines. The identity of the new compounds was established through FT-IR, ^1^H NMR, and ^13^C NMR analyses. Hydrazide (2), 1,3,4-oxadiazole derivative (3), and Mannich bases (**TG1-TG12**) represent new structures that have not been described in the literature.

The ^1^H NMR and ^13^C NMR spectra of **TG1-TG12** revealed the presence of a characteristic methylene group of the Mannich base as a singlet at δ∼4.6 ppm and δ∼69–70 ppm, respectively. Two methyl groups in the pyridothiazine-1,1-dioxide core were observed as two singlets at δ∼2.5 and δ∼2.7 ppm, and δ∼21,δ∼23 ppm, respectively. Moreover, pyridine hydrogen appeared at δ∼7.5 ppm in all Mannich base ^1^H NMR spectra between other phenyl hydrogen peaks.

### 2.2. Biological Tests

#### 2.2.1. Cyclooxygenase Inhibition

Table 1 presents the calculated percentage of inhibition of both COX-1 and COX-2 enzyme activity for the tested compounds at a concentration of 100 µM and selectivity ratios. Compounds **TG1**, **TG2**, **TG3**, **TG5**, **TG7**, and **TG8** displayed no inhibitory activity against either COX-1 or COX-2. Strong COX-2 inhibitory activity was observed after the use of **TG6** and, especially, **TG4**. **TG9** only inhibited the activity of the COX-1 enzyme. COX-1 selective activity for **TG10** was also observed. Compound **TG11**, as well as reference meloxicam, turned out to be a preferential COX-2 inhibitor. **TG12** was, in turn, a non-selective COX inhibitor.

Compounds exhibiting inhibitory activity on COX-1 and/or COX-2 (**TG4**, **TG6**, **TG9**, **TG10**, **TG11**, and **TG12**) were selected for the main stage of the study and included in an in vitro model of rheumatoid arthritis.

#### 2.2.2. Viability of Cell Cultures

**Figure 3:**
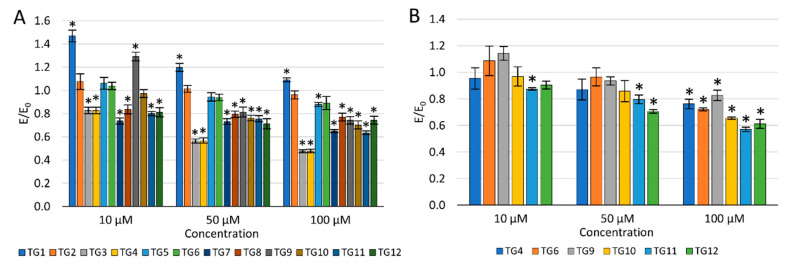
The effect of the tested compounds on the viability of normal human dermal fibroblasts (NHDF) cells (**A**) and human chondrocyte (TC28a2) cells (**B**); * *p* < 0.05—significant difference compared to the control.

The concentration dependence of the activity of the tested compounds was demonstrated—the viability of NHDF cells decreased with an increase in the concentration (Figure 3A). After incubation with the **TG1** compound in the entire concentration range and **TG9** at 10 µM, a statistically significant increase in proliferation was observed. Compounds **TG1**, **TG2**, and **TG6** exhibited no cytotoxic effect on normal NHDF fibroblasts in the whole range of concentrations tested—the cell viability was not lower than in the control culture (without the tested compounds). **TG4**, **TG7**, and **TG11** inhibited the proliferation of NHDF cells at all concentrations used. Compounds **TG8**, **TG10**, and **TG12** displayed a cytotoxic effect at concentrations of 50 and 100 µM, while compounds **TG3**, **TG5**, and **TG9** only significantly reduced cell growth at the highest concentration (100 µM).

As none of the tested compounds were cytotoxic (reduction of the fibroblast viability by more than 30%) at the lowest concentration of 10 µM, it was decided not to eliminate any of the compounds from further studies.

The impact on the viability of human chondrocytes (TC28a2) was only assessed for compounds displaying an inhibitory activity on COX-1 and/or COX-2 enzymes (Figure 3B). Compounds **TG9** and **TG12** showed no cytotoxic effect on these cells within the entire concentration range tested. However, compounds **TG4**, **TG6**, **TG10**, and **TG11** significantly inhibited the proliferation of chondrocytes at the highest concentration—100 µM.

#### 2.2.3. Reactive Oxygen Species and Nitric Oxide

**Figure 4:**
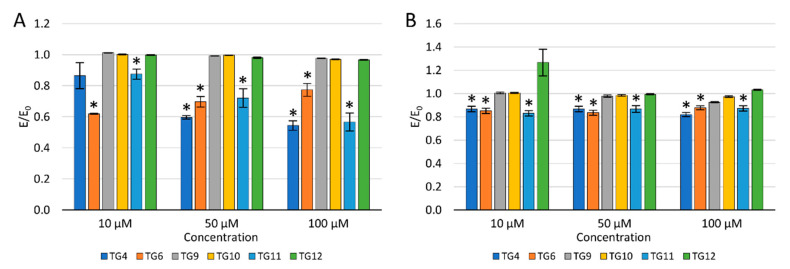
The effect of the tested compounds on the level of reactive oxygen species (**A**) and nitric oxide (**B**) in TC28a2 cells; * *p* < 0.05—significant difference compared to the control.

To evaluate the potential effect of selected compounds on inflammation, their impact on the levels of reactive oxygen species (ROS) and nitric oxide (NO) was checked (Figure 4).

After a 24 h incubation of the culture with the tested compounds, 100 µM H_2_O_2_ was added to evaluate the protective effect against oxidative stress. A decrease in the ROS level was observed after an earlier preincubation of chondrocytes (TC28a2) with **TG4**, **TG6**, and **TG11** compared to the control with H_2_O_2_. Simultaneously, a reduction in the ROS level was demonstrated for compound **TG10** at a concentration of 50 and 100 µM and **TG12**, only at a concentration of 100 µM. No reduction in the ROS level was observed after prior incubation with **TG9** within the entire concentration range tested.

Similarly, a decrease in the NO level was observed after the preincubation of chondrocytes with compounds **TG4**, **TG6**, and **TG11** within the entire range of tested concentrations. A statistically significant reduction in the NO level was also observed at a concentration of 100 µM for **TG9** and **TG12**. In turn, at a concentration of 10 µM of compound **TG12**, an increase in the NO level was observed.

#### 2.2.4. DNA Damage

**Figure 5:**
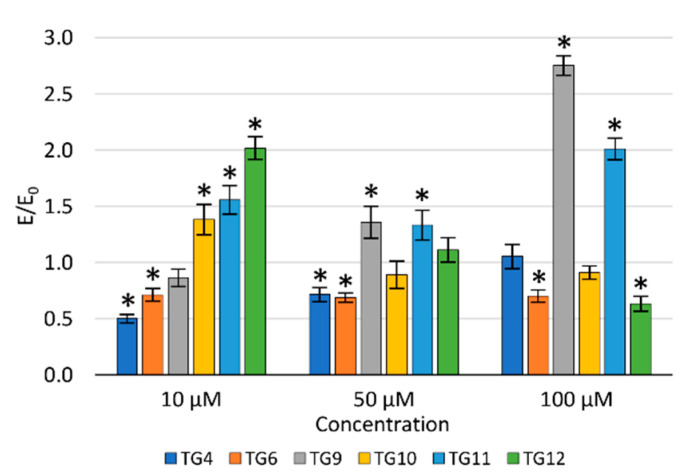
The effect of the tested compounds on the number of DNA double-strand breaks in TC28a2 cells; * *p* < 0.05—significant difference compared to the control.

The protective effect of the tested compounds against DNA damage caused by oxidative stress was assessed in the fast-halo assay (Figure 5). Based on the performed experiments, a statistically significant impact of compound **TG4** was demonstrated at a concentration of 10 µM, of compound **TG6** at 50 and 100 µM, and of compound **TG12** at 100 µM. For compounds **TG4**, **TG6**, and **TG9**, an increase in the number of double-strand DNA breaks was observed with an increase in the concentration. The opposite effect was demonstrated for the remaining compounds tested.

#### 2.2.5. Multiple-Criteria Decision Analysis

**Figure 6:**
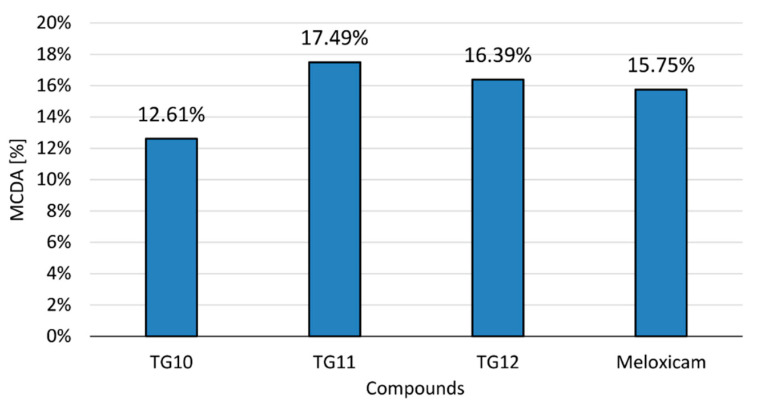
Multiple-criteria decision analysis (MCDA) of the anti-inflammatory effect of the tested compounds; MCDA was calculated based on the results of the cyclooxygenase inhibition assay and all in vitro tests on human chondrocytes that were performed.

The anti-inflammatory activity of the tested compounds at a concentration of 100 µM and the reference drug (meloxicam, 100 µM) was compared in the multiple-criteria decision analysis (MCDA), in which the results of the cyclooxygenase inhibition assay and all performed in vitro tests on human chondrocytes were summarized (Figure 6). All tests were assigned equal weights—they were of equal importance for the final result.

The results of MCDA demonstrated a positive anti-inflammatory effect of compounds **TG10**, **TG11**, and **TG12**. It can be assumed that **TG11** and **TG12** have stronger anti-inflammatory activity than meloxicam, with **TG11** having the best effect in the research model used.

### 2.3. Molecular Docking Study

In order to determine the binding mode and selectivity of the most active anti-COX compounds (**TG4**, **TG6**, and **TG10-TG12**), molecular docking was performed. In addition, the non-covalent intermolecular interactions of the compounds and both cyclooxygenases were characterized in detail. Due to the variety of high-resolution crystal structures of cyclooxygenase co-crystallized with ligands deposited in the Protein Data Bank, it is possible to analyze the binding manner of potential in detail for inhibitors [52]. In the present study, X-ray structures of COX-1 (PDB ID: 4O1Z) and COX-2 (PDB ID: 4M11), co-crystallized with the same ligand (meloxicam), were used during the molecular modeling procedure [53]. Structural studies have shown that both enzymes are characterized by nearly identical molecular weights and catalytic sites, although their amino acid sequence homology is only 65%. Due to the structural diversity, the ligands’ binding mode to the active site of isoforms is slightly different [54,55]. These subtle structural differences influence the selective inhibitor designing process. The presence of the smaller side chain of the Val523 residue in COX-2 results in an additional hydrophobic binding pocket as a target for selective anti-inflammatory agents. It was also evidenced that an increase in the affinity of COX-2 to ligands of a polar nature is caused by the presence of Arg513, instead of a His513 residue [56].

**Figure 7:**
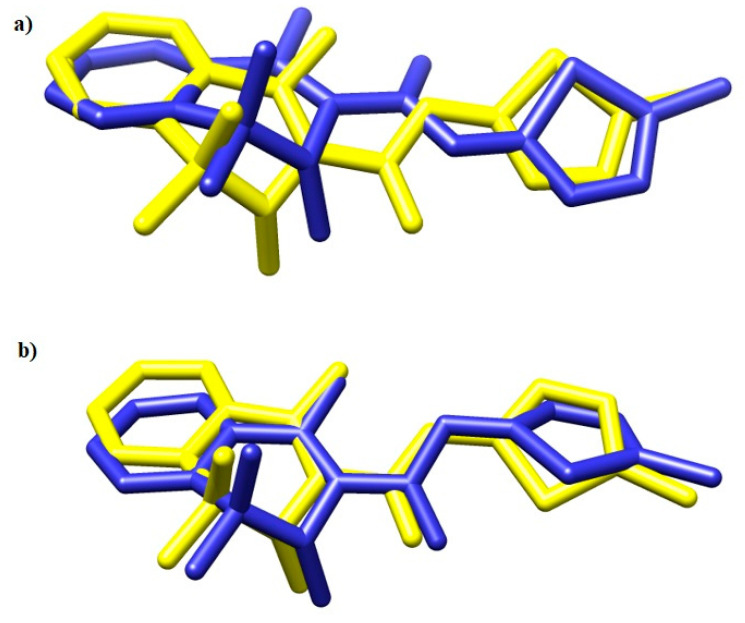
The overlay of meloxicam conformation in (**a**) 4M11 and (**b**) 4O1Z co-crystal (**yellow**) and the best docked conformer (**blue**) [53].

To validate the docking protocol, meloxicam (MXC) was docked into the crystal structures of COX-1 and COX-2. The results are presented in Figure 7.

The binding energies (kJ/mol) are presented in Table 2. **TG10** is the most effective COX-1 inhibitor (−46 kJ/mol), and **TG4** shows the greatest potency against the COX-2 isoform (−46 kJ/mol). In general, all investigated compounds (**TG4**, **TG6**, and **TG10-TG12**) are able to bind to the hydrophobic pockets of COX-1 and COX-2, surrounded by polar and hydrophobic amino acids. On the other hand, the binding mode affects the biological activity.

Compound **TG4**, for example, occupies the binding cavity of COX-2 in close proximity to Met113, Leu117, Leu352, Leu359, Trp387, Ala527, Ser530, and Leu533 amino acid residues, which represents the meloxicam mode of binding. As can be observed, the phenyl group can penetrate the hydrophobic side-pocket in the enzyme more deeply, due to the smaller valine residue in COX-2 compared to isoleucine in COX-1. Additionally, in this case, **TG4** revealed an inhibition profile similar to meloxicam, i.e., 41.60% and 44.95% for the synthesized derivative and MXC, respectively. The **TG4**–COX-2 complex is also stabilized by two hydrogen bonds created with Arg120 and Ser530. The **TG4** compound is biologically inactive under COX-1 binding conditions (Table 2). The energy of binding is positive and equal to 2.1 kJ/mol. The mode of binding differs from that in the active center of COX-2. In this case, **TG4** interacts with COX-1 via four hydrogen bonds with Arg120. The presence of the Ile523 residue and its interaction with the 5-tioxo-1,3,4-oxadiazole moiety influence the location of the trifluoromethylphenyl ring in a strongly polar and hydrophobic region composed of Phe205, Phe209, Phe381, Tyr348, and Tyr385 (Figures S1–S4).

Similar results were obtained for the **TG6** compound. As presented in Figures S5–S8, in COX-2, **TG6** shares almost the same position as meloxicam, although its selectivity is lower (the inhibition at a concentration of 100 μm is 24.43%). As can be observed, in the case of COX-1, the difluorophenyl ring is situated in close proximity to Phe205, Tyr348, Phe381, Phe381, Tyr385, and Ser530. This highly polar environment strongly determines the nature of interactions.

Compound **TG10** in the active center of COX-2 is stabilized via hydrogen bonds with Arg120 (bond length of 3.0Å and 3.4Å) and Tyr355 (bond length of 2.7 Å) and hydrophobic interactions with Val349, Leu359, Val523, Ala527, Ser530, and Leu531. On the other hand, **TG10** is essential for the COX-1 inhibitory potential. In this case, leucine 352, methionine 522, and glycine 526 residues interact directly with the nitrophenyl ring through hydrophobic interactions. It can be observed that in the COX-2 binding cavity, the nitrophenyl moiety is surrounded by Leu352, Val523, and Gly526 (Figures S9–S12).

**Figure 8:**
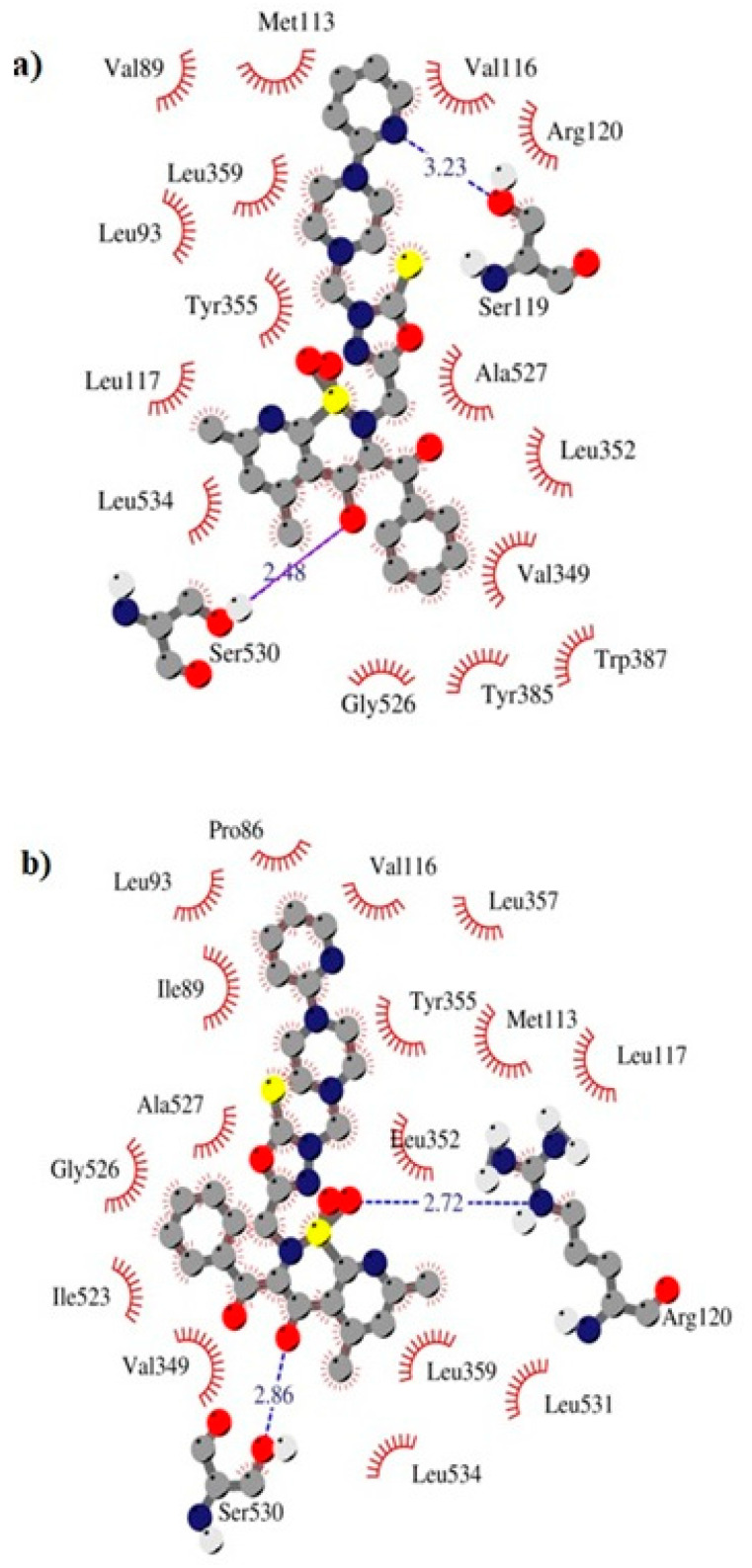
Intermolecular interactions between compound **TG11** and cyclooxygenase: (**a**) COX-2 and (**b**) COX-1.

**Figure 9:**
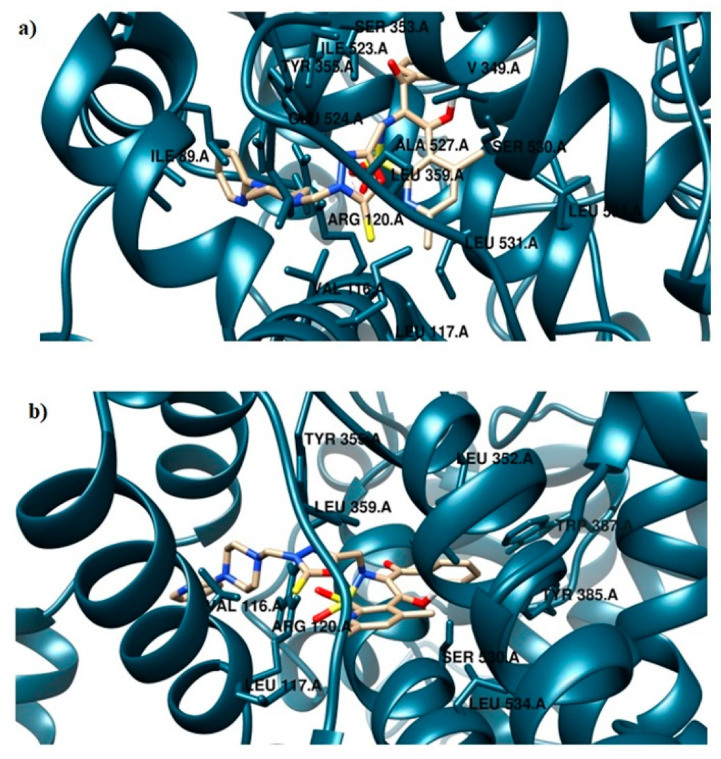
Binding mode of the analyzed compound: (**a**) **TG11**–COX-1 complex and (**b**) **TG11**–COX-2 complex.

The **TG11** compound might be a preferential and effective COX-2 inhibitor (Table 2). Under the COX-2 binding condition, compound **TG11** is involved in the hydrogen bonding with Ser530 (2.48Å), similar to meloxicam. There is also an extra weak hydrogen bond between the nitrogen of pyridine and oxygen of the Ser119 residue (3.23Å). In this case, the most stabilizing factor is the van der Waals interactions, which is strongly related to the binding mode of the compound in question. The phenyl ring of **TG11** is surrounded by aromatic residues of Tyr385 and Trp387 and glycine and valine of a hydrophobic nature (Figure 8a). A smaller selectivity might be observed under the COX-1 binding condition. The **TG11**–COX-1 complex is stabilized by two quite strong hydrogen bonds with Arg120 and Ser530 (bond length of 2.7Å and 2.9Å). A phenyl ring is situated in the area of the active site surrounded by residues, including Val349, Ile523, Gly526, and Ala627 (Figure 8b). The binding mode of complexes of **TG11** with COX-1 and COX-2 is presented in Figure 9.

Despite different modes of binding, compound **TG12** is not a selective inhibitor. In both cases, the free energy of binding is similar. The structural data are presented in Figures S13–S16.

## 3. Material and Methods

### 3.1. Chemistry

#### 3.1.1. Chemicals

All chemicals, reagents, and solvents used in the present study were purchased from commercial suppliers. Dry solvents were obtained according to the standard procedures. Progress of the reaction was monitored with the use of the thin-layer chromatography (TLC) technique on silica-gel-60-F254-coated TLC plates (Fluka Chemie GmbH) and visualized by UV light at 254 nm. The melting points of the synthesized compounds were determined with the use of an open capillary method on the Electrothermal Mel-Temp 1101D (Cole-Parmer, Vernon Hills, IL, USA) apparatus and were uncorrected. The ^1^H NMR (300 MHz) and ^13^C NMR (75 MHz) spectra were recorded on a Bruker 300 MHz NMR spectrometer (Bruker Analytische Messtechnik GmbH, Rheinstetten, Germany) in DMSO–*d_6_* using tetramethylsilane (TMS) as an internal reference. Chemical shifts (δ) are reported in ppm. The infrared (IR) spectra were determined on a Nicolet iS50 FT-IR spectrometer (Thermo Fisher Scientific, Waltham, MA, USA). Samples were applied as solids, and frequencies are reported in cm^−1^.

#### 3.1.2. Preparation and Experimental Properties of Compounds **2**, **3**, and **TG1-TG12**

##### Benzoyl-4-Hydroxy-5,7-Dimethyl-2*H*-Pyrido[3,2-*e*]-1,2-Thiazine-1,1-Dioxide-2-Acetic Acid Hydrazide 2

A solution of 3.7 g (9 mmol) of ester **1** in 35 mL ethanol was slightly warmed for about 10 min. The yellow suspension was cooled for a few minutes, and 4.5 mL (90 mmol, 10 eq.) of 80% hydrazine hydrate was then added. The precipitate dissolved immediately. The brown solution was refluxed for 5 h. The course of the reaction was controlled by TLC. The reaction was cooled, and the precipitate was filtered off and washed with ethanol. The crude product was purified by crystallization in ethanol with water.

Yield: 68%, m.p. 264–266 °C

^1^H NMR (DMSO-d_6_, 300 MHz) δ ppm: 2.54, 2.76 (2 × s, 6H, 2 × CH_3_), 3.91 (s, 2H, NH_2_), 4.08 (s, 2H, CH_2_), 7.47–7.49 (m, 2H, ArH), 7.56–7.61 (m, 2H, ArH + H-pyridine), 7.81–7.83 (m, 2H, ArH), 8.81 (s, 1H, NH), 13.81 (s, 1H, OH)

^13^C NMR (DMSO-d_6_, 75 MHz) δ ppm: 170.08, 166.06, 156.56, 151.43, 145,96, 129.84, 129.55, 129.04, 126.41, 118.84, 51.84, 23.91, 21.82

FT-IR (UATR, selected lines) ν_max_/cm^−1^: 3369 (NH), 3062 (NH_2_), 1688, 1589 (CO)

##### 3-Benzoyl-4-Hydroxy-5,7-Dimethyl-2-{[5-Tioxo-1,3,4-Oxadiazol-2-Ilo]Methyl}-2H-Pyrido[3,2-e]-1,2-Thiazine-1,1-Dioxide 3

A mixture of 0.2 g KOH (1.8 eq.) and 40 mL ethanol was stirred for a few minutes. Then, 0.804 g (2 mmol) hydrazide **2** was added to the solution and further mixed for 10 min. Subsequently, 2 mL CS_2_ (16 eq.) was added, and the mixture was refluxed for 4 h. On the completion of the reaction as monitored by TLC, the excess of CS_2_ and ethanol was removed under reduced pressure. The crude product was solubilized in a minimal quantity of water (about 1–2 mL). The mixture was acidified with 10% HCl until pH ~1. The resulting precipitated was filtered and washed with cold water. The crude product was purified in diethyl ether.

Yield: 91%, m.p. 235–238 °C decomp.

^1^H NMR (DMSO-d_6_, 300 MHz) δ ppm: 2.55, 2.73 (2 × s, 6H, 2 × CH_3_), 4.61 (s, 2H, CH_2_), 7.47–7.57 (m, 4H, ArH + H-pyridine), 7.80–7.83 (m, 2H, ArH), 14.08 (s, 1H, OH)

^13^C NMR (DMSO-d_6_, 75 MHz) δ ppm: 177.94, 158.13, 129.88, 126.16, 117.84, 46.64, 23.99, 21.56

FT-IR (UATR, selected lines) ν_max_/cm^−1^: 3324 (NH), 1595 (CO)

##### Mannich Bases **TG1-TG12**

A solution of 0.44 g (1 mmol) of **3**, 0.16 mL of 37% formaldehyde (*w/v*), and 1 mmol of appropriate piperazine in ethanol (30 mL) was stirred at room temperature for 3–5 h. The next day, the separated precipitate was filtered off and purified by crystallization in methanol.

##### 3-Benzoyl-4-Hydroxy-5,7-Dimethyl-2-{[4-((4-Phenyl-1-Piperazinyl)Methyl)-5-tioxo-1,3,4-Oxadiazol-2-Ilo]Methyl}-2*H*-Pyrido[3,2-*e*]-1,2-Thiazine-1,1-Dioxide **TG1**

Yield: 70%, m.p. 210–212 °C decomp.

^1^H NMR (DMSO-d_6_, 300 MHz) δ ppm: 2.53, 2.73 (2 × s, 6H, 2 × CH_3_), 2.76 (m, 4H, CH_2_-piperazine), 3.02 (m, 4H, CH_2_-piperazine), 4.63, 4.68 (2 × s, 4H, 2 × CH_2_), 6.75–6.80 (m, 2H, ArH), 6.91–6.94 (m, 2H, ArH), 7.18–7.23 (m, 2H, ArH), 7.47–7.58 (m, 3H, ArH + H-pyridine), 7.81–784 (m, 2H, ArH), 14.07 (s, 1H, OH)

^13^C NMR (DMSO-d_6_, 75 MHz) δ ppm: 177.98, 158.17, 151.49, 130.15, 129.93, 129.82, 129.38, 129.30, 126.22, 119.56, 117.68, 116.13, 69.84, 49.67, 48.64, 46.41, 23.96, 21.60

FT-IR (UATR, selected lines) ν_max_/cm^−1^: 1599 (CO)

##### 3-Benzoyl-4-Hydroxy-5,7-Dimethyl-2-{[4-((4-(4-Methylphenyl)-1-Piperazinyl)Methyl)-5-tioxo-1,3,4-Oxadiazol-2-Ilo]Methyl}-2*H*-Pyrido[3,2-*e*]-1,2-Thiazine-1,1-Dioxide **TG2**

Yield: 66%, m.p. 201–204 °C decomp.

^1^H NMR (DMSO-d_6_, 300 MHz) δ ppm: 2.18 (s, 3H, CH_3_-phenyl), 2.53, 2.73 (2 × s, 6H, 2 × CH_3_), 2.76 (m, 4H, CH_2_-piperazine), 2.96 (m, 4H, CH_2_-piperazine), 4.62, 4.67 (2 × s, 4H, 2 × CH_2_), 6.81–6.84 (m, 2H, ArH), 7.00–7.03 (m, 2H, ArH), 7.47–7.58 (m, 4H, ArH + H-pyridine), 7.81–7.84 (m, 2H, ArH), 14.06 (s, 1H, OH)

^13^C NMR (DMSO-d_6_, 75 MHz) δ ppm: 177.95, 158.15, 149.40, 130.16, 129.92, 129.82, 128.38, 127.38, 126.22, 121.02, 121.48, 118.99, 117.68, 116.38, 69.85, 49.68, 49.11, 46.39, 23.96, 21.60, 20.50

FT-IR (UATR, selected lines) ν_max_/cm^−1^: 1597 (CO)

##### 3-Benzoyl-4-Hydroxy-5,7-Dimethyl-2-{[4-((4-(2-Methoxyphenyl)-1-Piperazinyl)Methyl)-5-tioxo-1,3,4-Oxadiazol-2-ilo]Methyl}-2*H*-Pyrido[3,2-*e*]-1,2-Thiazine-1,1-Dioxide **TG3**

Yield: 53%, m.p. 183–185 °C decomp.

^1^H NMR (DMSO-d_6_, 300 MHz) δ ppm: 2.54, 2.75 (2 × s, 6H, 2 × CH_3_), 2.79 (m, 4H, CH_2_-piperazine), 2.86 (m, 4H, CH_2_-piperazine), 3.77 (s, 3H, OCH_3_), 4.59, 4.68 (2 × s, 4H, 2 × CH_2_), 6.85–6.92 (m, 4H, ArH), 7.58 (m, 4H, ArH + H-pyridine), 7.80 (m, 2H, ArH), 14.04 (s, 1H, OH)

^13^C NMR (DMSO-d_6_, 75 MHz) δ ppm: 178.29, 152.41, 141.54, 130.22, 129.94, 126.22, 123.03, 121.22, 118.50, 117.81, 112.19, 70.14, 55.68, 50.38, 49.97, 46.61, 23.94, 21.61

FT-IR (UATR, selected lines) ν_max_/cm^−1^: 1593 (CO)

##### 3-Benzoyl-4-Hydroxy-5,7-Dimethyl-2-{[4-((4-(3-Triflouomethylphenyl)-1-Piperazinyl)Methyl)-5-Tioxo-1,3,4-Oxadiazol-2-Ilo]Methyl}-2*H*-Pyrido[3,2-*e*]-1,2-Thiazine-1,1-Dioxide **TG4**

Yield: 58%, m.p. 193–196 °C decomp.

^1^H NMR (DMSO-d_6_, 300 MHz) δ ppm: 2.53, 2.72 (2 × s, 6H, 2 × CH_3_), 2.75 (m, 4H, CH_2_-piperazine), 3.13 (m, 4H, CH_2_-piperazine), 4.68 (2 × s, 4H, 2 × CH_2_), 7.06–7.24 (m, 4H, ArH), 7.42–7.58 (m, 4H, ArH + H-pyridine), 7.80–7.83 (m, 2H, ArH), 14.08 (s, 1H, OH)

^13^C NMR (DMSO-d_6_, 75 MHz) δ ppm: 177.90, 158.14, 151.69, 147.21, 130.43, 130.15, 129.92, 126.23, 123.07, 120.95, 119.53, 117.68, 115.32, 111.52, 69.76, 49.52, 48.01, 46.45, 23.93, 21.60

FT-IR (UATR, selected lines) ν_max_/cm^−1^: 1596 (CO)

##### 3-Benzoyl-4-Hydroxy-5,7-Dimethyl-2-{[4-((4-(2-Fluorophenyl)-1-Piperazinyl)Methyl)-5-tioxo-1,3,4-Oxadiazol-2-Ilo]Methyl}-2*H*-Pyrido[3,2-*e*]-1,2-Thiazine-1,1-Dioxide **TG5**

Yield: 77%, m.p. 214–216 °C decomp.

^1^H NMR (DMSO-d_6_, 300 MHz) δ ppm: 2.54, 2.74 (2 × s, 6H, 2 × CH3), 2.78 (m, 4H, CH_2_-piperazine), 2.91 (m, 4H, CH_2_-piperazine), 4.61, 4.68 (2 × s, 4H, 2 × CH_2_), 6.97–7.15 (m, 4H, ArH), 7.47–7.62 (m, 4H, ArH + H-pyridine), 7.81–7.84 (m, 2H, ArH), 14.07 (s, 1H, OH)

^13^C NMR (DMSO-d_6_, 75 MHz) δ ppm: 177.98, 158.11, 147.27, 130.17, 129.93, 127.35, 126.22, 125.29, 122.99, 121.02, 119.81, 117.75, 116.55, 116.28, 69.94, 50.43, 49.75, 46.56, 23.94, 21.62

FT-IR (UATR, selected lines) ν_max_/cm^−1^: 1598 (CO)

##### 3-Benzoyl-4-Hydroxy-5,7-Dimethyl-2-{[4-((4-(2,4-Difluorophenyl)-1-Piperazinyl)Methyl)-5-Tioxo-1,3,4-Oxadiazol-2-Ilo]Methyl}-2*H*-Pyrido[3,2-*e*]-1,2-Thiazine-1,1-Dioxide **TG6**

Yield: 66%, m.p. 205–208 °C decomp.

^1^H NMR (DMSO-d_6_, 300 MHz) δ ppm: 2.54, 2.74 (2 × s, 6H, 2 × CH_3_), 2.78 (m, 4H, CH_2_-piperazine), 2.86 (m, 4H, CH_2_-piperazine), 4.61, 4.66 (2 × s, 4H, 2 × CH_2_), 6.97–7.00 (m, 2H, ArH), 7.18 (m, 1H, ArH), 7.47–7.58 (m, 4H, ArH + H-pyridine), 7.81–7.84 (m, 2H, ArH), 14.05 (s, 1H, OH)

^13^C NMR (DMSO-d_6_, 75 MHz) δ ppm: 177.98, 158.15, 149.49, 130.15, 129.91, 126.21, 120.60, 117.74, 111.56, 69.91, 50.75, 49.75, 46.52, 23.94, 21.59

FT-IR (UATR, selected lines) ν_max_/cm^−1^: 1596 (CO)

##### 3-Benzoyl-4-Hydroxy-5,7-Dimethyl-2-{[4-((4-(4-Bromophenyl)-1-Piperazinyl)Methyl)-5-Tioxo-1,3,4-Oxadiazol-2-Ilo]Methyl}-2*H*-Pyrido[3,2-*e*]-1,2-Thiazine-1,1-Dioxide **TG7**

Yield: 59%, m.p. 179–182 °C decomp.

^1^H NMR (DMSO-d_6_, 300 MHz) δ ppm: 2.52, 2.72 (2 × s, 6H, 2 × CH_3_), 2.75 (m, 4H, CH_2_-piperazine), 3.03 (m, 4H, CH_2_-piperazine), 4.67 (2 × s, 4H, 2 × CH_2_), 6.87–6.90 (m, 2H, ArH), 7.26–7.35 (m, 2H, ArH), 7.47–7.58 (m, 4H, ArH + H-pyridine), 7.81–7.83 (m, 2H, ArH), 14.07 (s, 1H, OH)

^13^C NMR (DMSO-d_6_, 75 MHz) δ ppm: 178.09, 158.15, 150.59, 131.95, 130.16, 129.93, 126.21, 117.99, 117.67, 110.71, 69.77, 49.49, 48.26, 46.39, 23.96, 21.60

FT-IR (UATR, selected lines) ν_max_/cm^−1^: 1588 (CO)

##### 3-Benzoyl-4-Hydroxy-5,7-Dimethyl-2-{[4-((4-(2,4-Dichlorophenyl)-1-Piperazinyl)Methyl)-5-tioxo-1,3,4-Oxadiazol-2-Ilo]Methyl}-2*H*-Pyrido[3,2-*e*]-1,2-Thiazine-1,1-Dioxide **TG8**

Yield: 58%, m.p. 182–185 °C decomp.

^1^H NMR (DMSO-d_6_, 300 MHz) δ ppm: 2.53, 2.72 (2 × s, 6H, 2 × CH_3_), 2.72 (m, 4H, CH_2_-piperazine),3,08 (m, 4H, CH_2_-piperazine), 4.67 (2 × s, 4H, 2 × CH_2_), 6.95 (m, 1H, ArH), 7.11 (m, 1H, ArH), 7.37–7.40 (m, 1H, ArH), 7.47–7.54 (m, 4H, ArH + H-pyridine), 7.80–7.82 (m, 2H, ArH), 14.07 (s, 1H, OH)

^13^C NMR (DMSO-d_6_, 75 MHz) δ ppm: 178.68, 158.41, 151.17, 131.95, 130.91, 130.16, 129.92, 126.22, 120.28, 117.71, 116.85, 116.01, 70.06, 49.38, 47.89, 46.84, 23.95, 21.58

FT-IR (UATR, selected lines) ν_max_/cm^−1^: 1592 (CO)

##### 3-Benzoyl-4-Hydroxy-5,7-Dimethyl-2-{[4-((4-(2-cyanophenyl)-1-Piperazinyl)Methyl)-5-Tioxo-1,3,4-Oxadiazol-2-Ilo]Methyl}-2*H*-Pyrido[3,2-*e*]-1,2-Thiazine-1,1-Dioxide **TG9**

Yield: 75%, m.p. 200–203 °C decomp.

^1^H NMR (DMSO-d_6_, 300 MHz) δ ppm: 2.54 (s, 3H, CH_3_), 2.59 (m, 4H, CH_2_-piperazine),2.74 (s, 3H, CH_3_), 3.05(m, 4H, CH_2_-piperazine),4.59, 4.66 (2 × s, 4H, 2 × CH_2_), 7.08 (m, 3H, ArH), 7.58–7.67 (m, 6H, ArH + H-pyridine), 7.80 (m, 2H, ArH), 14.05 (s, 1H, OH)

^13^C NMR (DMSO-d_6_, 75 MHz) δ ppm: 178.29, 155.46, 149.48, 134.83, 130.17, 129.91, 126.23, 122.57, 119.55, 118.74, 117.92, 105.01, 69.98, 51.44, 49.81, 47.03, 23.97, 21.61

FT-IR (UATR, selected lines) ν_max_/cm^−1^: 2221 (CN), 1596 (CO)

##### 3-Benzoyl-4-Hydroxy-5,7-Dimethyl-2-{[4-((4-(4-Nitrophenyl)-1-Piperazinyl)Methyl)-5-tioxo-1,3,4-Oxadiazol-2-Ilo]Methyl}-2*H*-Pyrido[3,2-*e*]-1,2-Thiazine-1,1-Dioxide **TG10**

Yield: 74%, m.p. 195–198 °C decomp.

^1^H NMR (DMSO-d_6_, 300 MHz) δ ppm: 2.52, 2.70 (2 × s, 6H, 2 × CH_3_), 3.36 (m, 8H, CH_2_-piperazine), 4.65 (s, 4H, 2 × CH_2_), 7.01–7.04(m, 2H, ArH), 7.46–7.58 (m, 5H, ArH + H-pyridine), 7.79–7.81 (m, 1H, ArH), 8.03–8.06 (m, 2H, ArH), 14,06 (s, 1H, OH)

^13^C NMR (DMSO-d_6_, 75 MHz) δ ppm: 178.09, 155.18, 149.44, 147.19, 137.54, 130.13, 129.90, 126.20, 126.15, 117.75, 113.25, 69.64, 49.32, 46.61, 46.53, 23.96, 21.55

FT-IR (UATR, selected lines) ν_max_/cm^−1^: 1596 (CO)

##### 3-Benzoyl-4-Hydroxy-5,7-Dimethyl-2-{[4-((4-(2-Pyridyl)-1-Piperazinyl)Methyl)-5-Tioxo-1,3,4-Oxadiazol-2-Ilo]Methyl}-2*H*-Pyrido[3,2-*e*]-1,2-Thiazine-1,1-Dioxide **TG11**

Yield: 58%, m.p. 197–202 °C decomp.

^1^H NMR (DMSO-d_6_, 300 MHz) δ ppm: 2.42–2.48 (m, 8H, CH_2_-piperazine),2.52, 2.70 (2 × s, 6H, 2 × CH_3_), 4.66 (2 × s, 4H, 2 × CH_2_), 6.61–6.65 (m, 1H, pyridine), 6.80–6.83 (m, 1H, pyridine), 7.46–7.59 (m, 6H, ArH + H-pyridine), 7.80–7.82 (m, 1H, pyridine), 8.11 (m, 1H, pyridine), 14.06 (s, 1H, OH)

^13^C NMR (DMSO-d_6_, 75 MHz) δ ppm: 178.09, 159.41, 158.41, 147.99, 147.16, 137.99, 130.14, 129.91, 126.19, 121.02, 117.72, 113.63, 107.65, 69.96, 49.52, 46.46, 44.85, 23.96, 21.58

FT-IR (UATR, selected lines) ν_max_/cm^−1^: 1592 (CO)

##### 3-Benzoyl-4-Hydroxy-5,7-Dimethyl-2-{[4-((4-(2-Pyrimidyl)-1-Piperazinyl)Methyl)-5-Tioxo-1,3,4-Oxadiazol-2-Ilo]Methyl}-2*H*-Pyrido[3,2-*e*]-1,2-Thiazine-1,1-Dioxide **TG12**

Yield: 74%, m.p. 200–204 °C decomp.

^1^H NMR (DMSO-d_6_, 300 MHz) δ ppm: 2.39 (m, 4H, CH_2_-piperazine), 2.52, 2.69 (2 × s, 6H, 2 × CH_3_), 3.63 (m, 4H, CH_2_-piperazine), 4.64 (s, 4H, 2 × CH_2_), 6.62 (m, 1H, pyrimidine), 7.51 (m, 4H, ArH + H-pyridine), 2.77 (m, 2H, ArH),8.35–8.36 (m, 2H, pyrimidine), 14.05 (s, 1H, OH)

^13^C NMR (DMSO-d_6_, 75 MHz) δ ppm: 178.29, 161,61, 158.38, 130.11, 129.89, 129.77, 126.18, 117.77, 110.73, 70.05, 49.54, 46.52, 43.45, 23.92, 21.55

FT-IR (UATR, selected lines) ν_max_/cm^−1^: 1585 (CO)

### 3.2. Biological Section

#### 3.2.1. Cell Line

The in vitro tests were performed on two human cell lines—the normal human dermal fibroblasts (NHDF) and human chondrocytes (TC28a2) purchased from ATCC (Manassas, VA, USA). Both cell lines were grown in a CO_2_-incubator (at 37 °C in 5% CO_2_ and 95% humidity). The cell morphology was assessed at least twice a week, and the cell culture was passaged or the medium was changed. The NHDF cells were cultivated in DMEM without phenol red and TC28a2 cells in DMEM with phenol red. Both media were supplemented with 10% fetal bovine serum (FBS), 2 mM l-glutamine, 1.25 µg/mL amphotericin B, and 100 µg/mL gentamicin. The media were stored at 4–8 °C for up to a month.

#### 3.2.2. Tested Compounds

The 10 mM stock solutions of the tested compounds in DMSO were prepared and stored at −20 °C for up to 6 months. For the in vitro studies, stock solutions were dissolved in an appropriate culture medium to final concentrations of 10, 50, and 100 µM, so the DMSO concentration did not reach 1%.

#### 3.2.3. Experimental Design

In the first stage of the study, the viability of the cells was assessed after 48 h-long incubation with the tested compounds in two cell lines. A simple model of rheumatoid arthritis was designed for compounds with COX-1 and/or COX-2 inhibitory activity. In the remaining experiments, after 24 h-long incubation of the cell cultures with the tested compounds, the supernatant was removed. The cultures were washed, and 100 µM H_2_O_2_ solution was added for 1 h to induce oxidative stress (characteristic of inflammation, i.e., in rheumatoid arthritis).

#### 3.2.4. Sulforhodamine B (SRB) Assay

The cytotoxicity of the tested compounds on NHDF and TC28a2 cells was assessed in the sulforhodamine B (SRB) assay. The cells were seeded at a density of 10,000 cells per well and incubated for 24 h to allow cells to adhere to the well surface. The medium was subsequently removed and the test compounds were added at concentrations of 10–100 µM (prepared immediately before being added to the culture) for the next 48 h. The 10% *w*/*v* cold TCA solution was added to plates for 1 h at 4–8 °C to fix the cells. The culture plates were washed four times with running water. The cell cultures were air-dried at room temperature (RT), and the SRB solution (0.4% dye solution in 1% acetic acid) was then added for 30 min at RT. The unbound dye was rinsed with 1% (*v*/*v*) acetic acid. The plates were air-dried again at RT, and 10mM Trizma base was added to dissolve protein-bound dye for 30 min. Finally, the absorbance was measured at 565 nm using a Varioskan LUX microplate reader (Thermo Fisher Scientific, Waltham, MA, USA). Based on the obtained results, the percentage of cell viability after incubation with each of the tested compounds in relation to the control was calculated.

#### 3.2.5. Reactive Oxygen Species and Nitric Oxide

The DCF-DA and Griess assays were performed to assess ROS and NO levels, respectively. The 2′,7′-dichlorofluorescein diacetate (DCF-DA) solution was prepared immediately before use by dissolving DCF-DA in 100% ethanol and adjusting it to a working concentration of 10 µM in deionized water. After 24 h of incubation with the tested compounds, the cells were washed, and 100 µM H_2_O_2_ was added for 1 h to induce oxidative stress. The supernatant was subsequently removed from the plates with cells, the cultures were washed again, and the DCF-DA solution was added for another 1 h. Simultaneously, after the incubation with H_2_O_2_, the medium was transferred in a volume of 50 µM to new culture plates. The plates with DCF-DA solution were analyzed with the Varioskan LUX microplate reader (λ_ex_ = 485 nm and λ_em_ = 535 nm). Components A and B from the Griess Reagent Kit (cat. no. G7921; Thermo Fisher Scientific, Waltham, MA, USA) were added to plates with supernatant and the plates were left for 30 min in the dark at RT. Finally, the absorbance was measured using Varioskan LUX (λ = 548 nm).

#### 3.2.6. Fast-Halo Assay

After incubating the cells with the administered compounds, the supernatant was transferred to previously prepared tubes. Next, 0.1% Tryple solution in PBS was added to the plates for 5 min at 37 °C to detach the cells from the surface of the wells. After this time, the cells were transferred to appropriate tubes and centrifuged for 5 min at 1000× *g*. The supernatant was then removed, and the culture was washed with PBS and centrifuged again under the same conditions. Finally, the cells were suspended at a density of 1000 cells/µL in PBS with Ca^2+^ and Mg^2+^. The tubes were placed in a water bath set to 37 °C. A 1.25% low gelling point agarose solution was added to each test tube, and this solution was placed on a base slide covered with a high gelling point agarose solution and covered with a coverslip. The slide prepared in this way was placed on a cooling block for 10 min for gelling. After this time, the coverslip was removed, and the slide was placed in a lysis buffer overnight at 4–8 °C. The next day, the slides were transferred to a buffer with pH = 13 for 30 min at RT in the dark, and the preparations were then washed twice for 5 min in a neutralizing buffer. The slides were finally stained with a 5 µM DAPI solution for 20 min and assessed under a fluorescence microscope. Pictures of 50 randomly selected cells were taken for each slide.

#### 3.2.7. Cyclooxygenase Inhibition Assay

A ready-to-use kit (cat. no. 701050; Cayman Chemical Company, MI, USA) was used to evaluate the COX peroxidase activity for all tested compounds (at a concentration of 100 µM). The peroxidase activity measurement was performed after 2 min of incubation at RT, with the use of Varioskan LUX (λ = 590 nm). Based on the absorbance obtained, the COX inhibition percentage was calculated separately for COX-1 and COX-2 enzymes after incubation for 2 min with the tested compounds. The selectivity of cyclooxygenase inhibition was also calculated as a ratio (%inh. COX-2/%inh. COX-1). Meloxicam was used as a reference compound.

#### 3.2.8. Statistical Analysis

All presented results are E/E_0_ ratios expressed as the mean ± SEM (standard error of the mean), where E stands for the result for the culture with the addition of the tested substance, and E_0_ stands for the control without the compound. Statistical significance was calculated compared to the control using a one-way ANOVA test (with a Tukey post-hoc test). In all tests, the level of significance was *p* < 0.05. Multiple-criteria decision analysis (MCDA) was used to summarize the results in the rheumatoid arthritis model and compare the effect of each tested compound, as previously described [57].

### 3.3. Molecular Modeling

Structure optimization of the new derivatives was performed at the B3LYP/6-31G(d,p) level of theory by evaluating the Hessian matrix to confirm that the geometries corresponded to the minima on the potential energy surface [58,59,60,61]. The water solution was taken into account using a polarizable continuum model (PCM) [62,63]. All optimizations were performed by applying the Gaussian 09 package [64].

Molecular docking simulations were performed using the AutoDock4.2 package [65]. The atomic coordinates of chain A of cyclooxygenases were used as an input. Water molecules and all co-factors were removed. Polar hydrogens, Gasteiger charges, and solvent parameters were added. The binding site was defined using a grid of 90 × 90 × 90 points with a grid space of 0.375 Å. The center of the box was located on the active site according to crystallized inhibitor coordination. The Lamarckian genetic algorithm with a local search was employed for a total of 100 runs for each binding site. In each calculation, populations of 150 individuals with 27,000 generations and 250,000 energy evaluations were adopted. The estimated binding free energy (Δ*G_binding_*) allows one to evaluate the affinity of a ligand–protein complex and can be expressed by the following formula:

Δ*G_binding_* = [Δ*G_intermolecular_* + Δ*G_internal_* + Δ*G_tors_*] − Δ*G_unbound_*. (1)

The intermolecular interaction energy (Δ*G_intermolecular_*) is the sum of van der Waals, hydrogen bonding, desolvation, and electrostatic terms between the inhibitor and the binding site of the protein.

Δ*G_intermolecular_* = [Δ*G_vdw_* + Δ*G_hbond_* + Δ*G_desolv_*] + Δ*G_el_* (2)

The Chimera visualization program was used to present the obtained results [66]. Schematic 2D diagrams were prepared using the academic version of LIGPLOT v. 4.5.3 software shared by the European Bioinformatics Institute (EMBL-EBI), Cambridge, UK [67].

## 4. Conclusions

This study presents a biological evaluation of a new series of Mannich base-type hybrid compounds containing an arylpiperazine residue, 1,3,4-oxadiazole ring, and pyridothiazine-1,1-dioxide core. Their in vitro anti-COX-1/COX-2, antioxidant, and cytotoxic effect on normal NHDF fibroblasts was studied. A molecular docking study was performed to understand the binding interaction of the compounds at the active site of cyclooxygenases. COX-2 inhibitory activity was observed after the use of **TG6** and, especially, **TG4**. **TG9** only inhibited the activity of the COX-1 enzyme. COX-1 selective activity for **TG10** was also observed. Compound **TG11**, as well as reference meloxicam, turned out to be a preferential COX-2 inhibitor. **TG12** was shown to be the least selective among all compounds.

It is worth noting that in the test of NHDF cells’ viability, the compounds **TG1**, **TG2**, and **TG6** exhibited no cytotoxic effect on normal fibroblasts in the whole range of concentrations tested. Compounds **TG8**, **TG10**, and **TG12** displayed a cytotoxic effect at concentrations of 50 and 100 µM, while compounds **TG3**, **TG5**, and **TG9** only significantly reduced cell growth at the highest concentration (100 µM). It should be noted that none of the tested compounds were cytotoxic (reduction of the fibroblast viability by more than 30%) at the lowest concentration of 10 µM. In the test evaluating the viability of human chondrocytes (TC28a2), compounds **TG9** and **TG12** showed no cytotoxic effect on these cells within the entire concentration range tested. Additionally, **TG4**, **TG6**, **TG10**, and **TG11** only significantly inhibited the proliferation of chondrocytes at the highest concentration of 100 µM.

To evaluate the potential effect of selected compounds on inflammation, their impact on the levels of reactive oxygen species (ROS) and nitric oxide (NO) was checked. A decrease in the ROS level was observed after an earlier preincubation of chondrocytes with **TG4**, **TG6**, and **TG11** compared to the control with H_2_O_2_. Simultaneously, a reduction in the ROS level was demonstrated for compound **TG10** at a concentration of 50 and 100 µM, and for **TG12**, only at a concentration of 100 µM. Similarly, a decrease in the NO level was observed after the preincubation of chondrocytes with compounds **TG4**, **TG6**, and **TG11** in the entire range of the tested concentrations. A statistically significant reduction in the NO level was also observed at a concentration of 100 µM for **TG9** and **TG12**. The protective effect of the tested compounds against DNA damage caused by oxidative stress was assessed in the fast-halo assay. Based on the performed experiments, a statistically significant impact of compound **TG4** was demonstrated at a concentration of 10 µM, of **TG6** at concentrations of 50 and 100 µM, and of **TG12** at 100 µM.

Finally, the anti-inflammatory activity of both the tested compounds at a concentration of 100 µM and the reference drug meloxicam was compared in multiple-criteria decision analysis (MCDA), in which the results of the cyclooxygenase inhibition assay and all performed in vitro tests on human chondrocytes were summarized. The results of MCDA revealed a positive anti-inflammatory effect of compounds **TG10**, **TG11**, and **TG12**. It can be assumed that **TG11** and **TG12** have stronger anti-inflammatory activity than meloxicam, with **TG11** having the best effect in the research model used.

In order to determine the binding mode and the selectivity of the most active anti-COX compounds (**TG4**, **TG6**, and **TG10**-**TG12**), molecular docking was performed. In general, all investigated compounds were able to bind to the hydrophobic pockets of COX-1 and COX-2, surrounded by polar and hydrophobic amino acids. On the other hand, it was shown that differences in the structure of compounds affect the binding mode and biological activity.

## Figures and Tables

**Table 1:** Cyclooxygenase (COX) inhibition calculated for COX-1 and COX-2 enzymes after incubation for 2 min with the tested compounds at a concentration of 100 µM and the COX selectivity ratio.

Compound	COX Inhibition at a Concentration of 100 μM		COX Selectivity Ratio %inh. COX-2/%inh. COX-1
	COX-1	COX-2	
TG1	N/A	N/A	-
TG2	N/A	N/A	-
TG3	N/A	N/A	-
TG4	N/A	41.60%	-
TG5	N/A	N/A	-
TG6	N/A	24.43%	-
TG7	N/A	N/A	-
TG8	N/A	N/A	-
TG9	1.20%	N/A	-
TG10	42.79%	10.05%	0.23
TG11	12.21%	27.52%	2.25
TG12	31.93%	31.38%	0.98
Meloxicam	30.33%	44.95%	1.48

**Table 2:** Binding energies to COX-1 and COX-2 of the most active anti-COX compounds (**TG4**, **TG6**, and **TG10-TG12)**.

Compound	COX Inhibition at a Concentration of 100 μM		COX-1 Free Energy of Binding (kJ/mol)	COX-2 Free Energy of Binding (kJ/mol)
	COX-1	COX-2		
TG4	N/A	41.60%	2.1	−46.5
TG6	N/A	24.43%	13.3	−44.4
TG10	42.79%	10.05%	−46.5	−41.4
TG11	12.21%	27.52%	−38.4	−45.2
TG12	31.93%	31.38%	−45.6	−45.2
Meloxicam	30.33%	44.95%	-	-

## Supplementary Materials

The following are available online at https://www.mdpi.com/1422-0067/21/23/9122/s1.
