# Supplementary material for: New 1,3,4-Oxadiazole Derivatives of Pyridothiazine-1,1-Dioxide with Anti-Inflammatory Activity
Source: Int J Mol Sci. 2020 Nov 30;21(23):9122. doi: 10.3390/ijms21239122 (PMC7729791; doi:10.3390/ijms21239122)
Supplement: Supplementary file 1 [file ijms-21-09122-s001.pdf]

International Journal of
Molecular Sciences

Supplementary Material

In the figures, which show the intermolecular interactions between investigated compounds and cyclooxygenases, hydrophobic interactions are marked in blue and hydrogen bonds are marked in red.

In the figures, which show the binding mode of analyzed compounds, some ribbons were removed to expose the interactions.

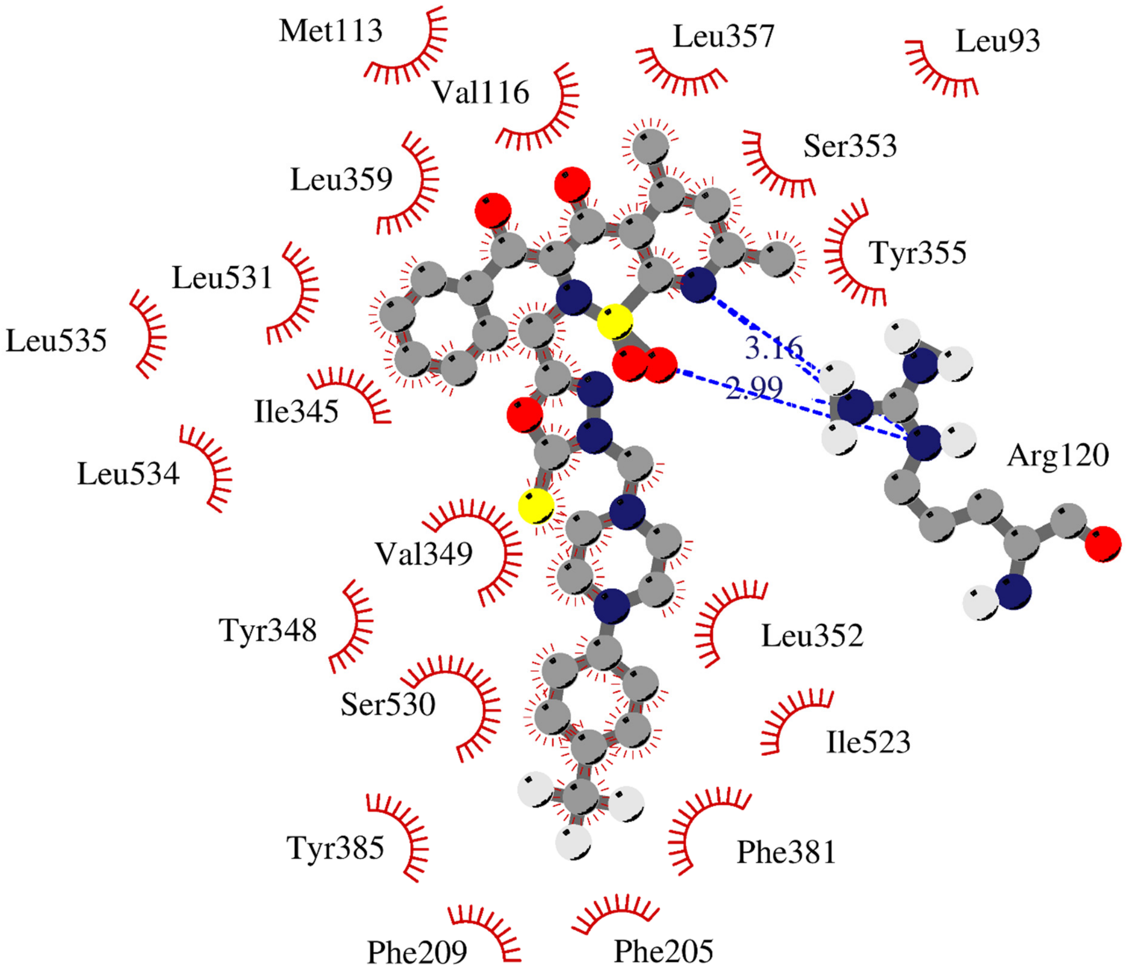

Figure S1. The intermolecular interactions of the investigated compound **TG4** and cyclooxygenase (COX)-1.

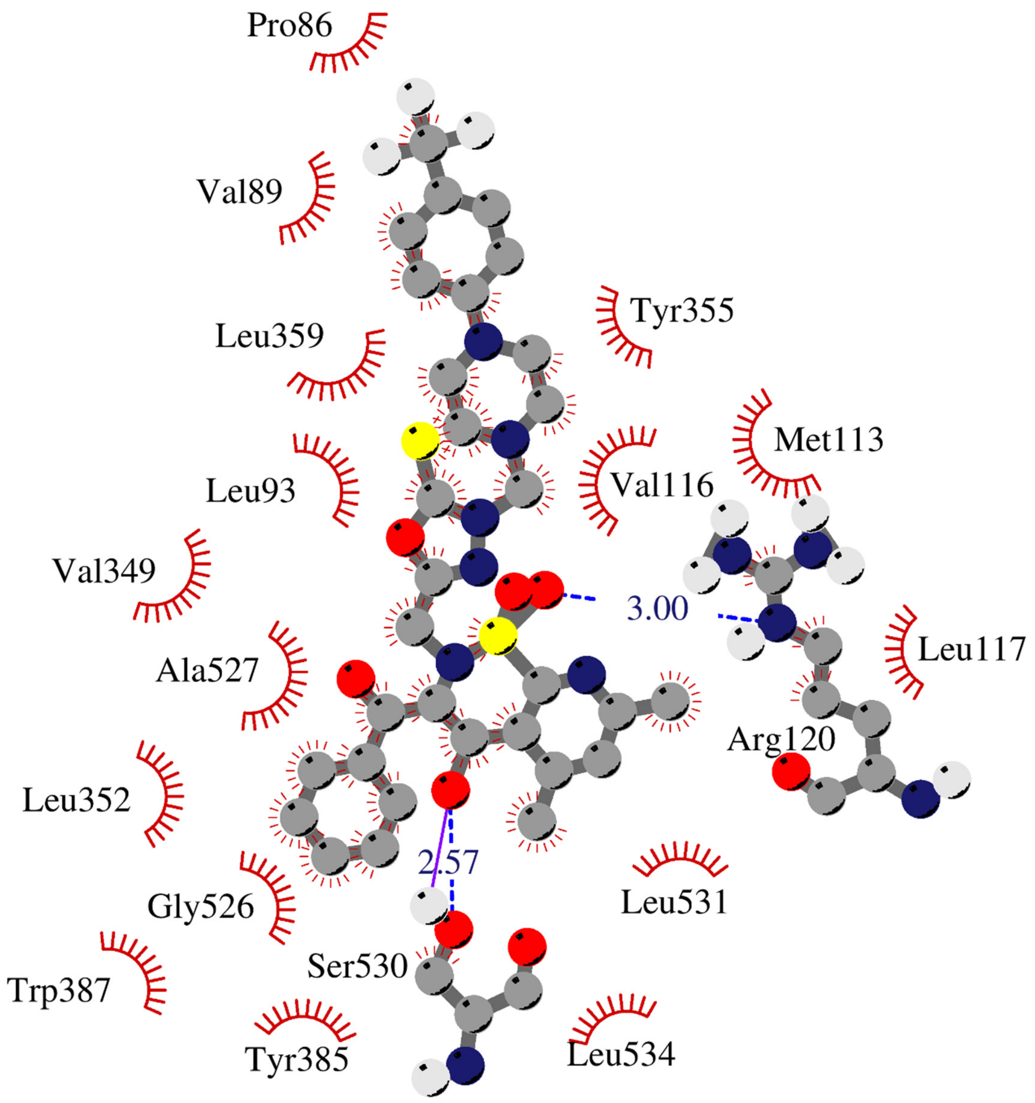

Figure S2. The intermolecular interactions between the investigated compound **TG4** and cyclooxygenase COX-2.

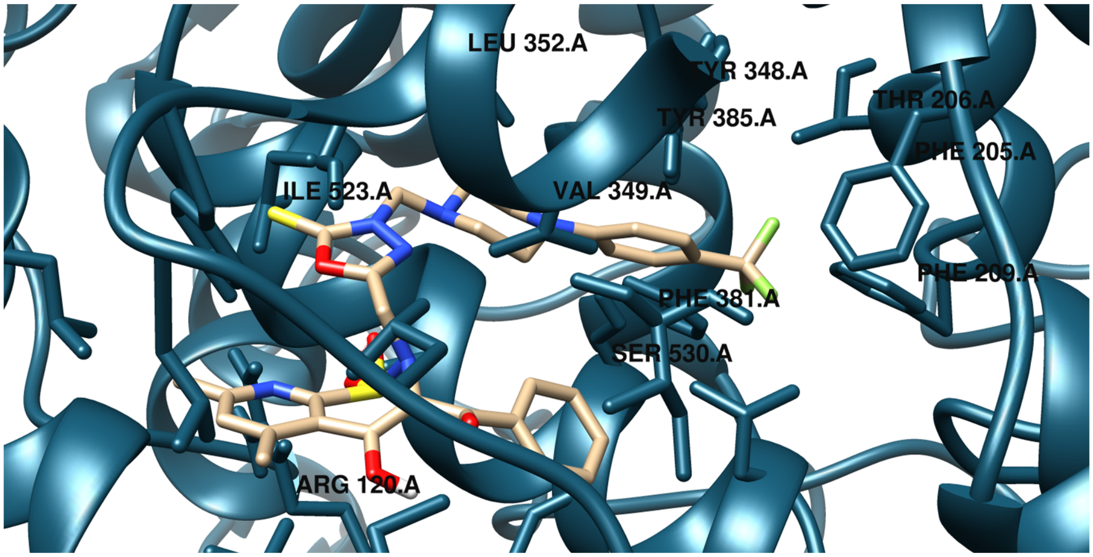

Figure S3. The binding mode of the analyzed compound **TG4**–COX-1 complex.

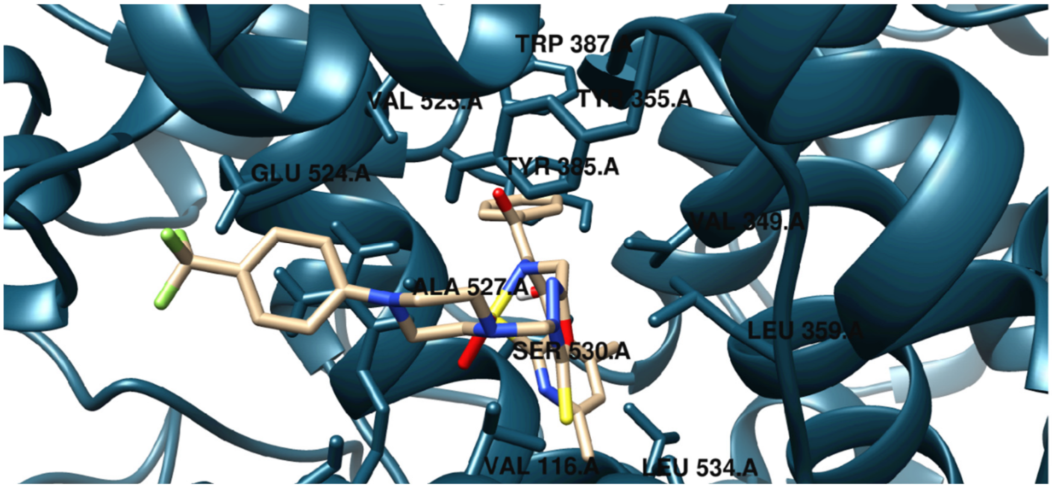

Figure S4. The binding mode of the analyzed compound **TG4**–COX-2 complex.

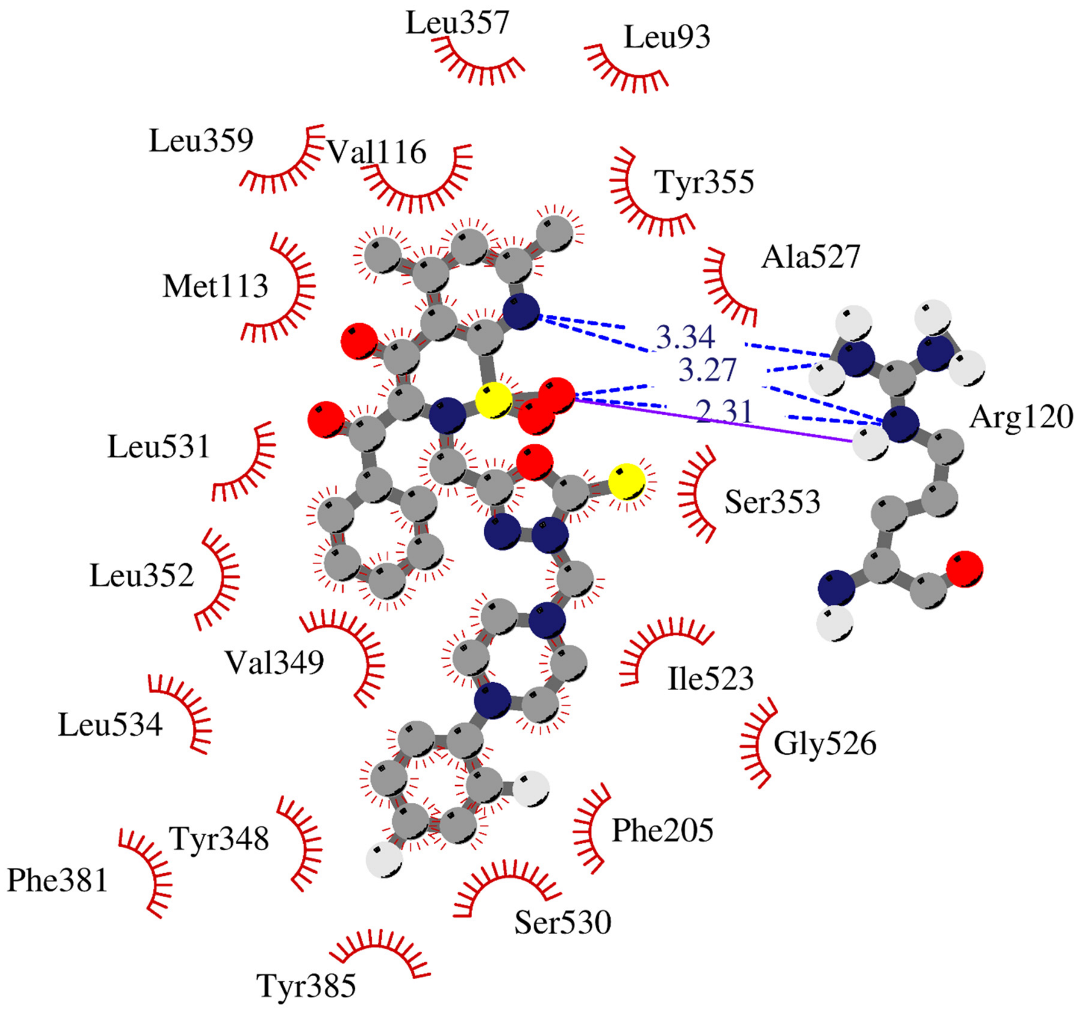

Figure S5. The intermolecular interactions between the investigated compound **TG6** and cyclooxygenase COX-1.

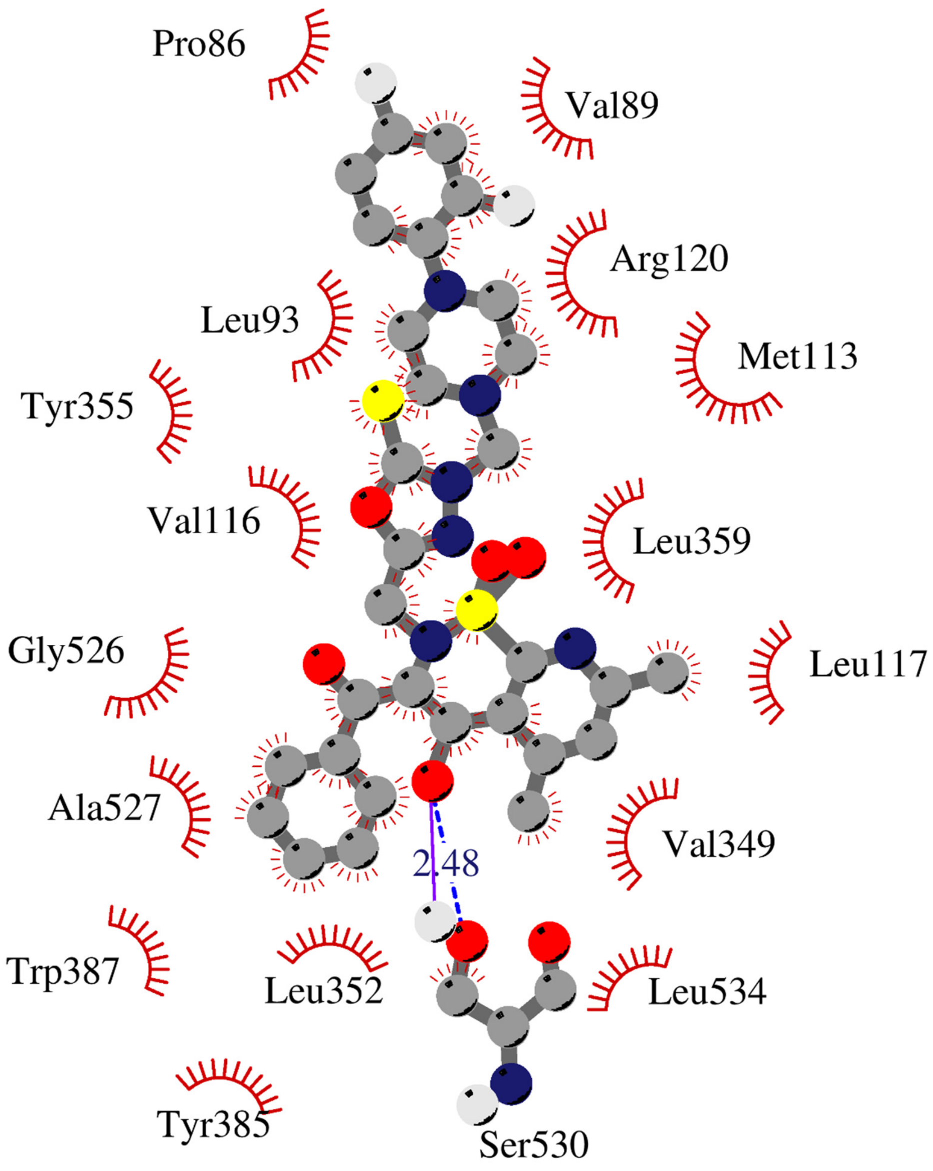

Figure S6. The intermolecular interactions between the investigated compound **TG6** and cyclooxygenase COX-2.

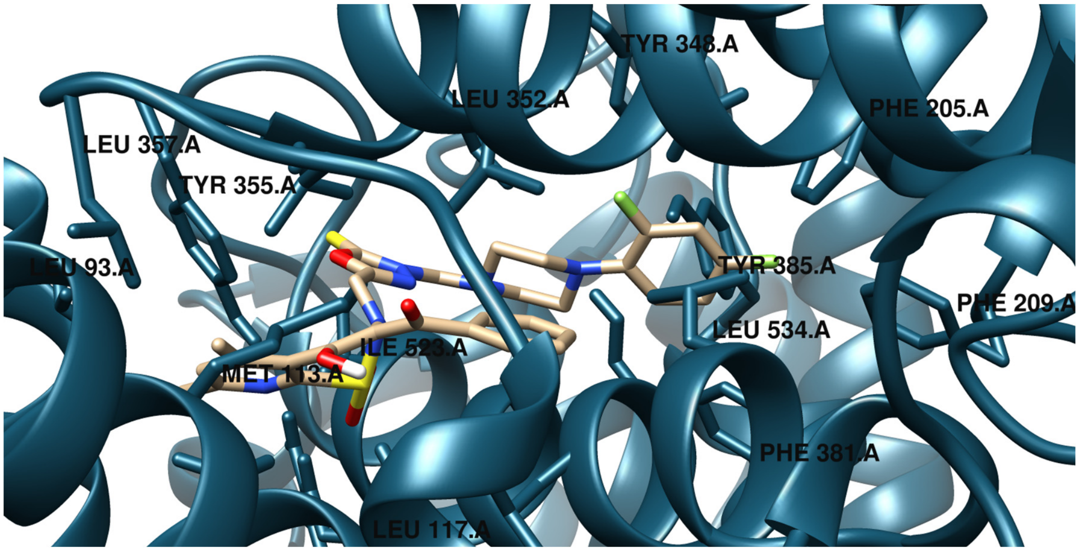

Figure S7. The binding mode of the analyzed compound **TG6**–COX-1 complex.

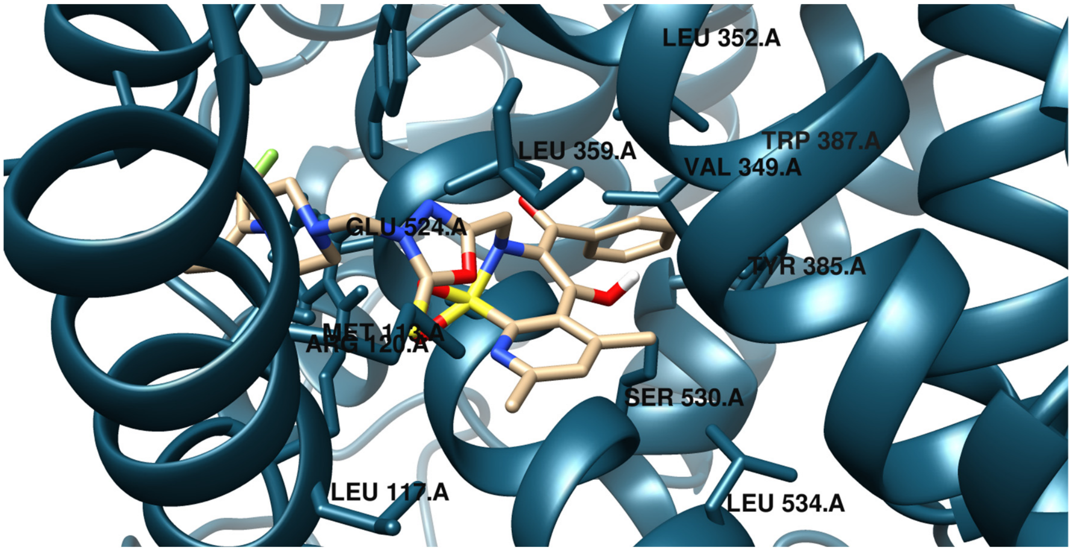

Figure S8. The binding mode of the analyzed compound **TG6**–COX-2 complex.

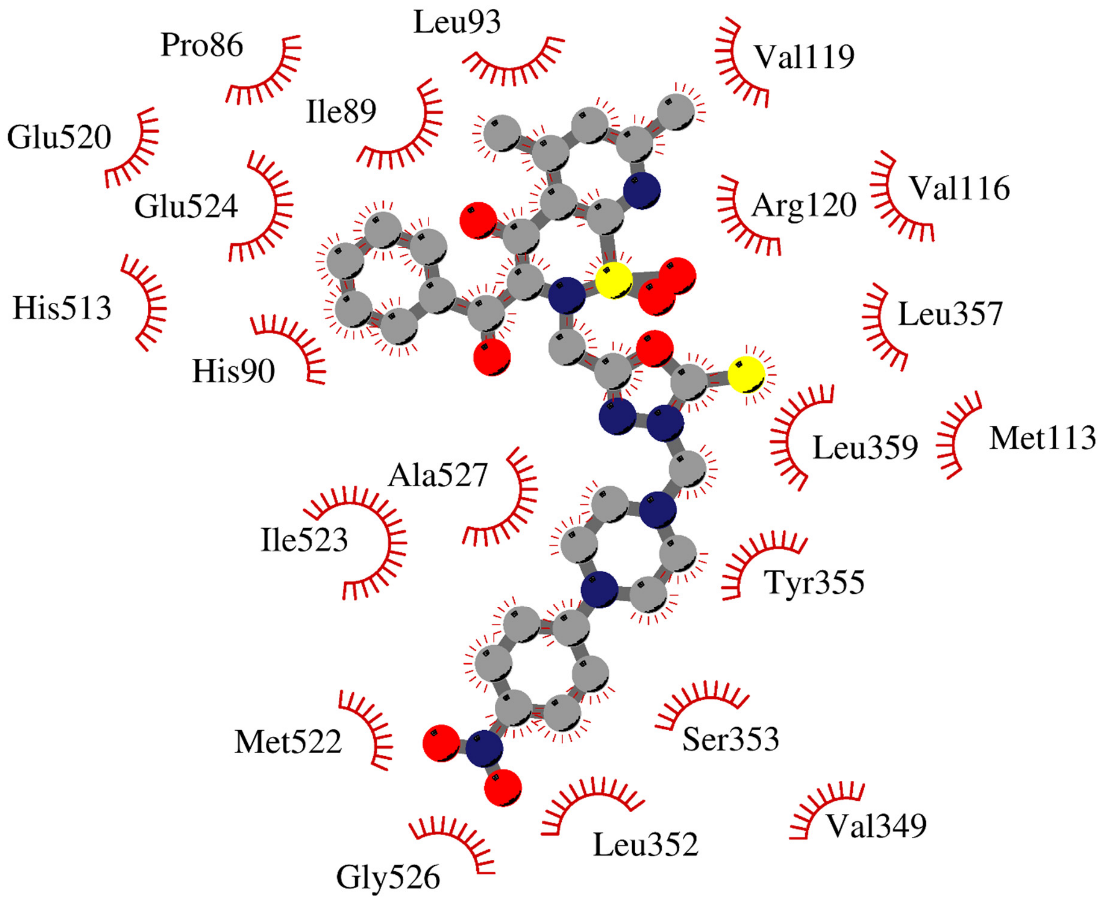

Figure S9. The intermolecular interactions between the investigated compound **TG10** and cyclooxygenase COX-1.

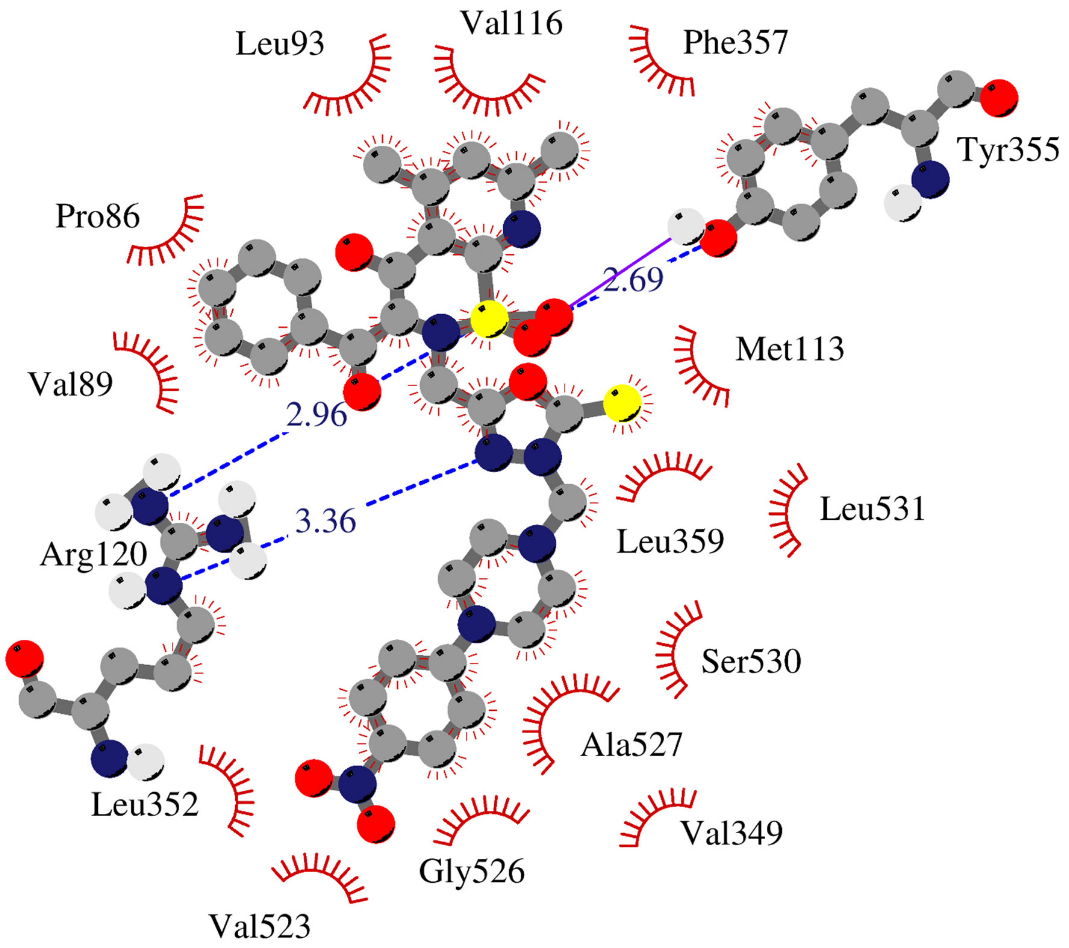

Figure S10. The intermolecular interactions between the investigated compound **TG10** and cyclooxygenase COX-2.

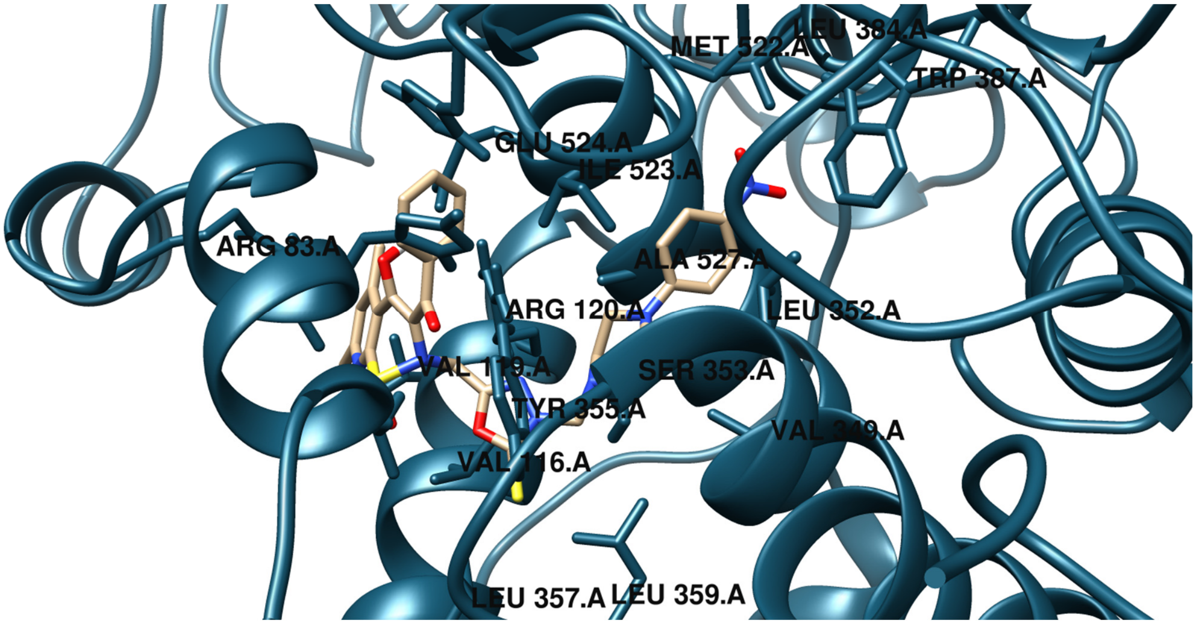

Figure S11. The binding mode of the analyzed compound **TG10**–COX-1 complex.

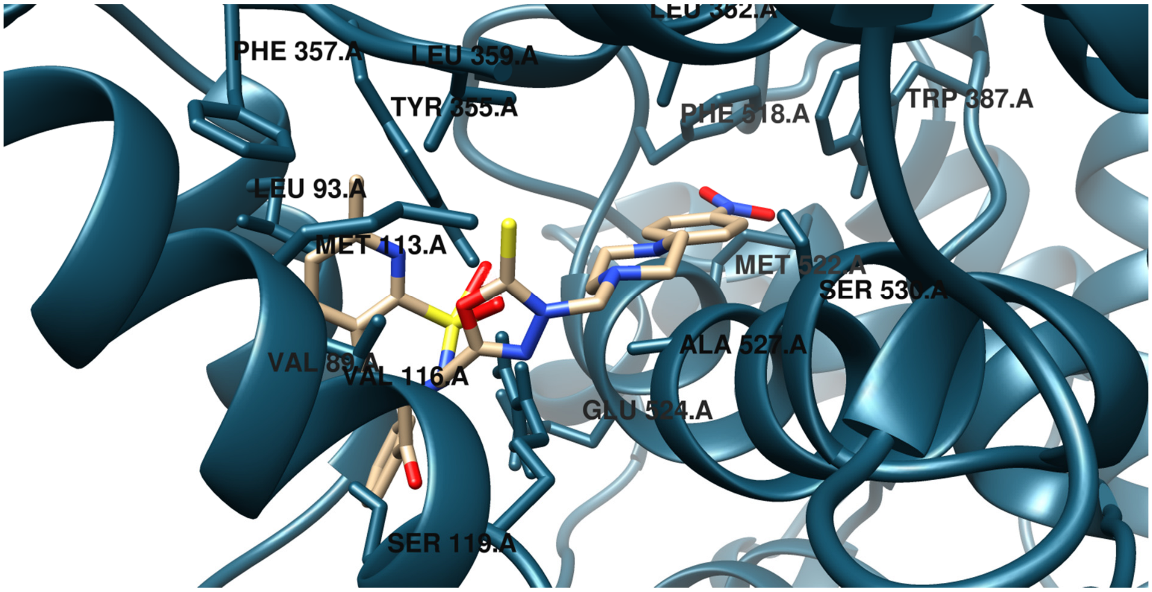

Figure S12. The binding mode of the analyzed compound **TG10**–COX-2 complex.

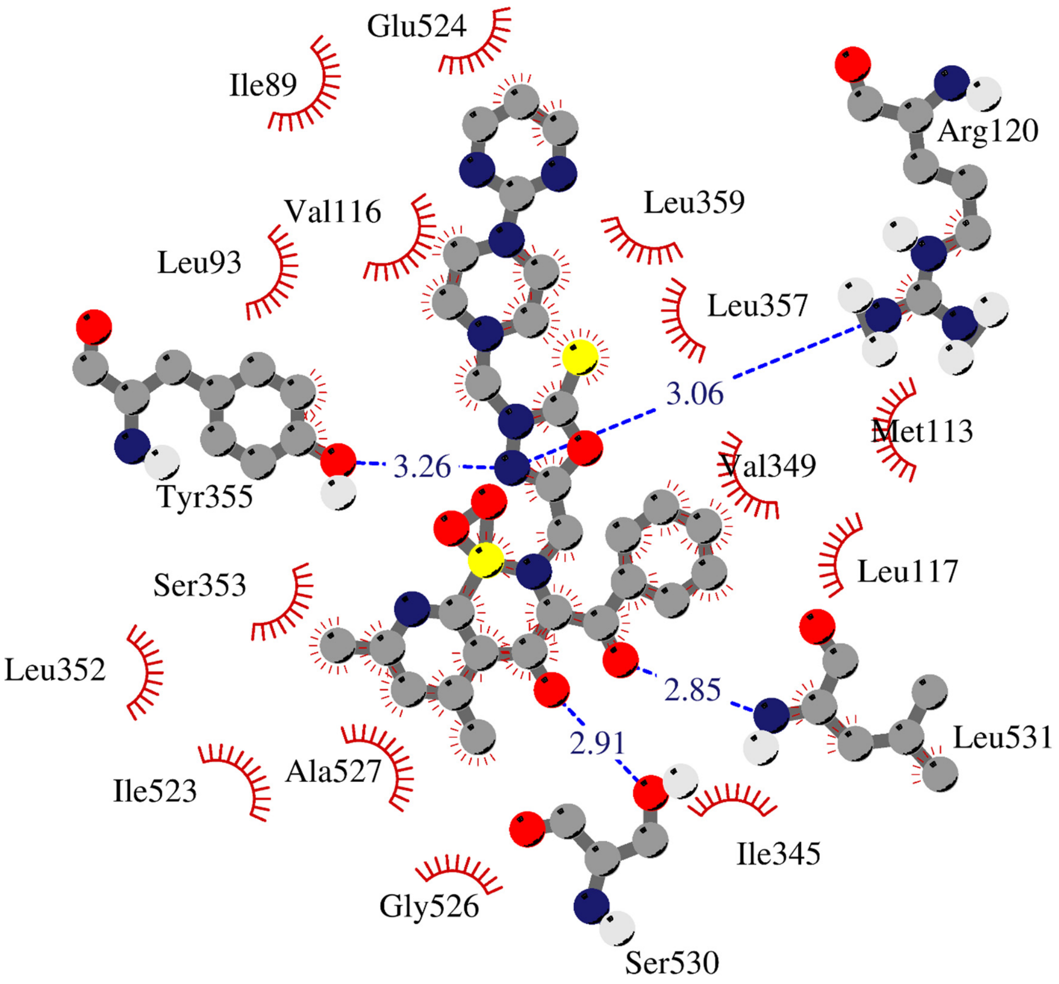

Figure S13. The intermolecular interactions between the investigated compound **TG12** and cyclooxygenase COX-1.

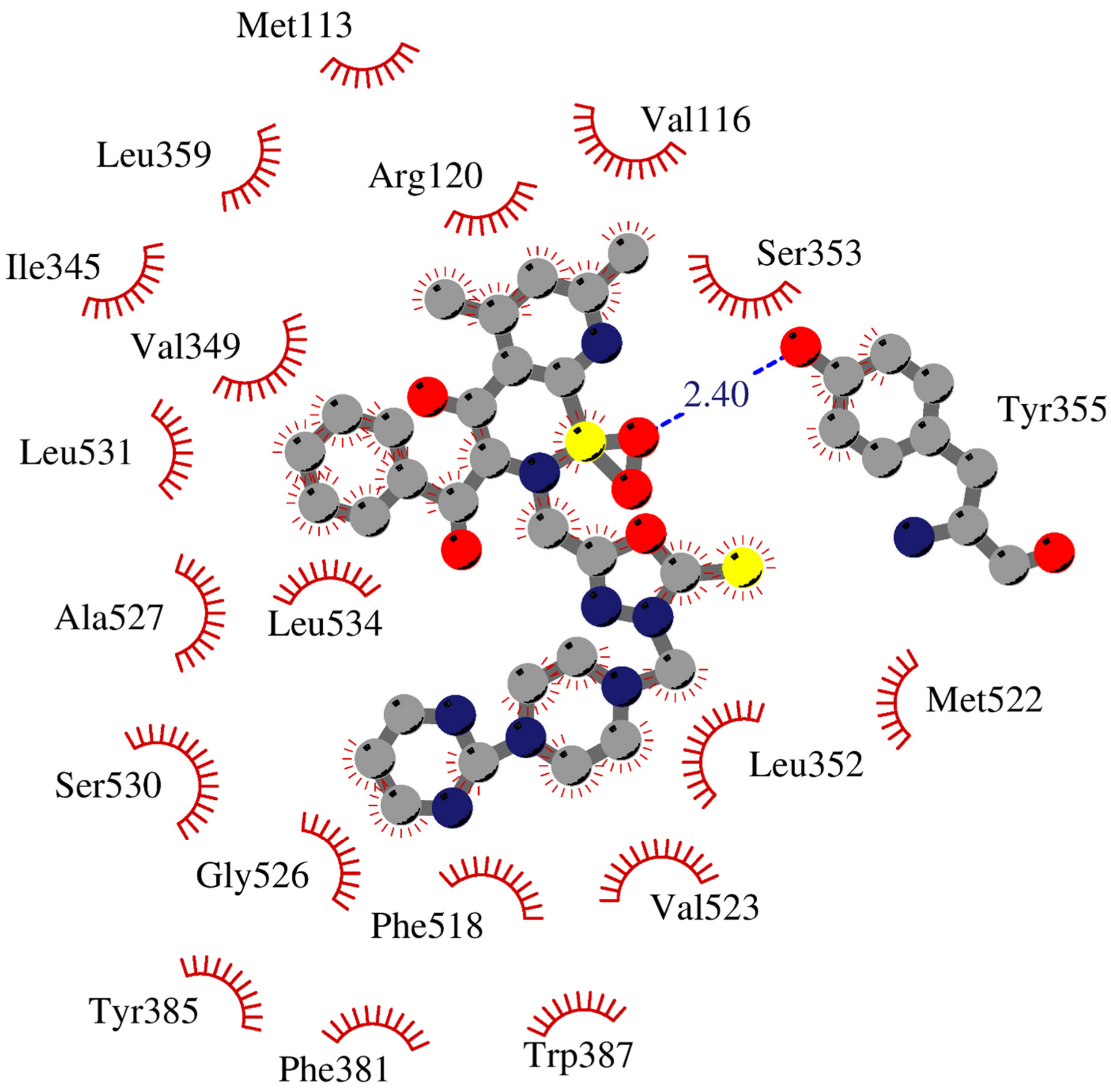

Figure S14. The intermolecular interactions between the investigated compound **TG12** and cyclooxygenase COX-2.

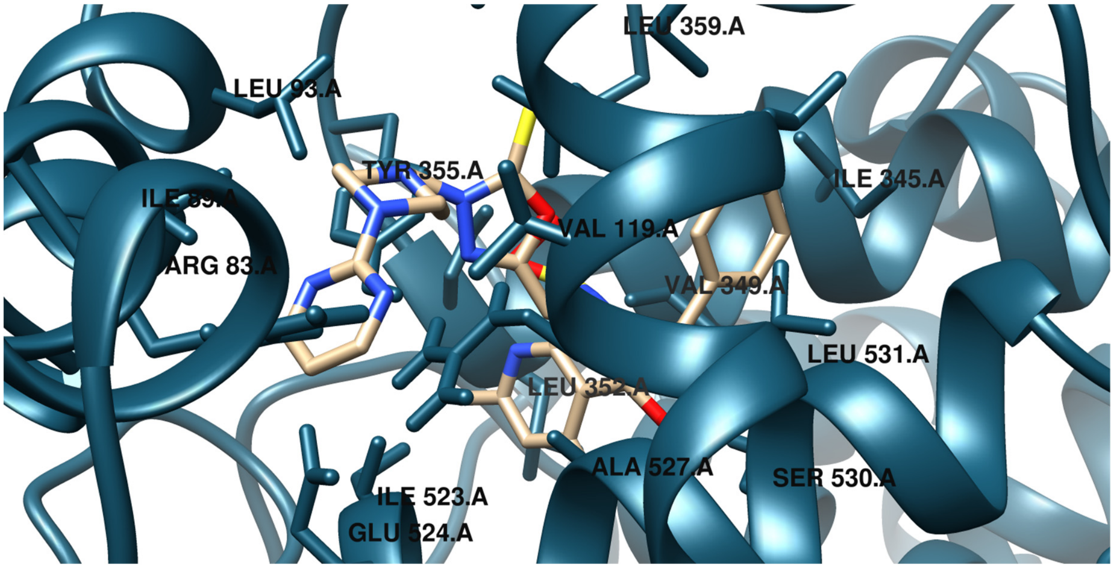

Figure S15. The binding mode of the analyzed compound **TG12**–COX-1 complex.

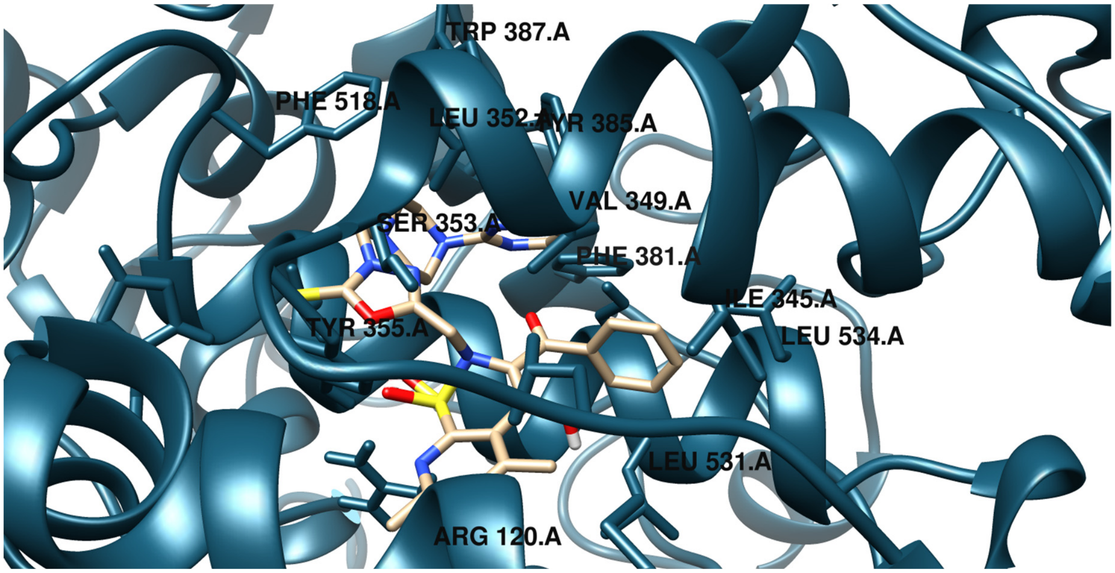

Figure S16. The binding mode of the analyzed compound TG12–COX-2 complex.
